# Allostery through DNA drives phenotype switching

**DOI:** 10.1038/s41467-021-23148-2

**Published:** 2021-05-20

**Authors:** Gabriel Rosenblum, Nadav Elad, Haim Rozenberg, Felix Wiggers, Jakub Jungwirth, Hagen Hofmann

**Affiliations:** 1 Department of Chemical and Structural Biology, Rehovot, Israel; 2 Department of Chemical Research Support, Rehovot, Israel; 3 Department of Chemical and Biological Physics, Weizmann Institute of Science, Rehovot, Israel

**Keywords:** Single-molecule biophysics, Cryoelectron microscopy

## Abstract

Allostery is a pervasive principle to regulate protein function. Growing evidence suggests that also DNA is capable of transmitting allosteric signals. Yet, whether and how DNA-mediated allostery plays a regulatory role in gene expression remained unclear. Here, we show that DNA indeed transmits allosteric signals over long distances to boost the binding cooperativity of transcription factors. Phenotype switching in *Bacillus subtilis* requires an all-or-none promoter binding of multiple ComK proteins. We use single-molecule FRET to demonstrate that ComK-binding at one promoter site increases affinity at a distant site. Cryo-EM structures of the complex between ComK and its promoter demonstrate that this coupling is due to mechanical forces that alter DNA curvature. Modifications of the spacer between sites tune cooperativity and show how to control allostery, which allows a fine-tuning of the dynamic properties of genetic circuits.

## Abstract (web summary)

Most insights on DNA-mediated allostery upon transcription factor (TF) binding were either based on artificial promoters or found to be short-ranged. Here authors use single-molecule FRET and cryo-EM to show that Bacillus subtilis bacteria utilize long-range allostery in a stochastic and reversible phenotype switch.

## Introduction

**Fig. 1:**
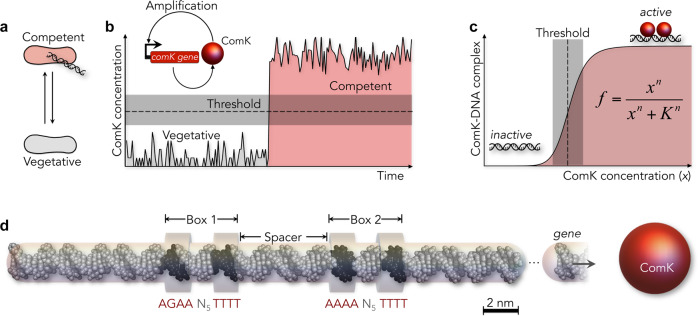
Schematics describing the model for phenotype switching in *B. subtilis*. **a** *B. subtilis* can reversibly switch to a competent phenotype (red). **b** Switching is triggered by copy number fluctuations of ComK (grey) that, once stochastically exceeding a threshold (dashed line), cause its auto-amplification to high concentrations (red). Inset: scheme of the auto-amplification of ComK. **c** The threshold (grey) requires a cooperative DNA binding of ComK, modelled by the Hill equation with cooperativity *n*, affinity *K*, and fraction of complexes *f*. **d** ComK promoters consist of two boxes separated by a spacer. Here, the *comG* promoter with an 18 bp spacer and an imperfect A-tract in box 1 is shown. For size comparison, a ComK monomer (Stokes radius: 2.07 ± 0.03 nm, Supplementary Fig. 1) is shown as red sphere.

Allostery is the structural coupling between ligand sites in biomolecules. Binding of a ligand to one site facilitates or hampers the binding of a second ligand to a distant site^1–5^. The resulting cooperativity regulates the activity of many proteins and molecular machines^6–8^, but it is also key for the behaviour of genetic circuits with binary^9^, oscillatory^10^, excitable^11,12^, or pulsing^13^ dynamics. The past decades have seen growing evidence that allostery is also an inherent property of DNA^14–27^, which has far reaching consequences for our understanding of promoter sequences. Yet, most insights on DNA-mediated allostery upon transcription factor (TF) binding were either based on artificial promoters^14,28^ or found to be short-ranged^19,20,25^. Whether natural promoters evolved to efficiently transmit allosteric signals across many nanometres remained largely unclear. Here, we show that *Bacillus subtilis* bacteria utilize long-range allostery in a stochastic and reversible phenotype switch (Fig. 1a). In the competent phenotype, *B. subtilis* can take up DNA from the medium^11,29^. The master regulator of the switch from vegetative to competent cells is the TF ComK^11,12^. The model postulates a positive feedback regulation of *comK* gene expression once ComK levels stochastically surpass a critical threshold (Fig. 1b)^30^. The critical threshold acts like an analogue-to-digital converter: the ComK target promoter is inactive at low concentrations, but it switches cooperatively to an active state within a narrow ComK concentration range (Fig. 1c)^11,12^. Yet, how the promoter mediates cooperative ComK binding is unknown. Using single-molecule Förster resonance energy transfer (smFRET) and cryo-electron microscopy (cryo-EM) we show how ComK binding at one site enhances binding to a distant site via allosteric changes in DNA.

## Results

### Distant binding sites communicate

**Fig. 2:**
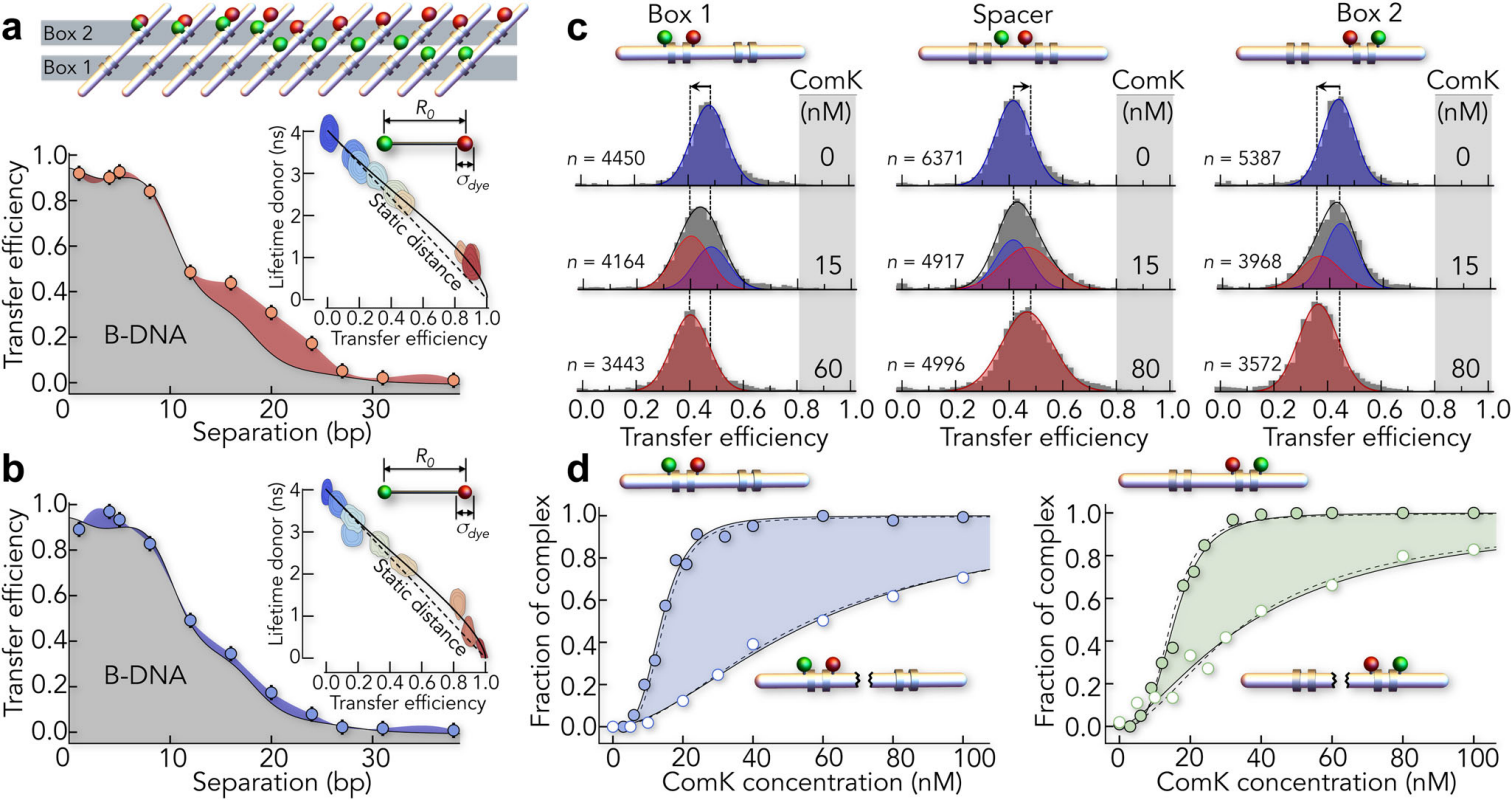
DNA-binding cooperativity of ComK probed with smFRET. **a**, **b** Mapping of distances in box 2 with FRET at higher (**a** pH 7) and lower (**b** pH 4) persistence length. Grey region indicates the expected dependence for B-DNA^34^. Insets: fluorescence lifetimes of the donor as function of the transfer efficiency. Dashed line is the expectation for a single distance and solid line is a fit with a Gaussian distance distribution to account for dye flexibility with widths of 0.76 and 0.66 nm for pH 7 and pH 4, respectively (see ‘Methods’). The dimension of the dye clouds is schematically indicated compared to the Förster distance *R*_0_ = 5.4 nm. **c** SmFRET histograms of the *comG* promoter (93 bp) with an 18 bp spacer indicate a change in DNA on ComK binding. FRET is probed in box 1 (left), in the spacer (middle), and in box 2 (right). Solid lines (black) are fits with superpositions of two Gaussian peaks for the free (blue) and ComK-bound (red) promoter. **d** Fraction of the ComK–DNA complexes probed in box 1 (left) and box 2 (right) in the presence (filled circles) and absence (empty circles) of the respective other box. The single-box constructs are 43 bp in length. Lines are fits with the Hill equation (solid) and with a mechanistic binding model (dashed, ‘Methods’ Eqs. (20) and (21)).

ComK target promoters consist of two elements (box 1 and box 2, hereafter) separated by spacer sequences of variable length (Fig. 1d)^31^. Three spacers of 8, 18, and 31 base pairs (bp) are known in the ComK response genes *addAB*, *comG*, and *comK*, respectively^31^. Each box in a promoter contains an adenine–thymine (AT)-rich sequence (Fig. 1d). Such A-tracts are ubiquitous throughout all kingdoms of life, presumably due to their potential to curve DNA^32,33^. To assess the structural properties of the promoter, we engineered a set of *comG* promoters (18 bp spacer) that we site-specifically labelled with donor and acceptor fluorophores for confocal smFRET measurements (Supplementary Fig. 2 and Supplementary Table 1). When we compared the experimental FRET efficiencies with theoretical values calculated for extended B-type DNA^34^, we noted substantial deviations across one box, suggesting an overall curved topology (Fig. 2a). Given that DNA curvature scales with DNA stiffness^35^, which is sensitive to the protonation state of the phosphate backbone, we repeated the experiment under more acidic conditions (pH 4.0, Fig. 2b). Indeed, we obtained reduced FRET values that showed a better apparent agreement with the calculated B-DNA profile. This result indicated that the *comG* promoter is curved at neutral pH. Importantly, the correlation between donor fluorescence lifetimes and FRET values is close to the expectation from Förster theory (see ‘Methods’), which indicates that the structure of the *comG* promoter does not fluctuate substantially at the length scale probed by FRET (Fig. 2a,b inset). Yet, small-amplitude motions cannot be ruled out and require a more sophisticated analysis to be identified^36^.

To probe ComK binding cooperativity, we labelled the *comG* promoter with a FRET-pair either at each box or within the spacer (Fig. 2c). In the absence of ComK, we find FRET values between 0.4 and 0.5. Upon addition of ComK, the FRET peaks shift to lower values for box 1 and box 2, equivalent to a distance increase, whereas the FRET efficiency increased for the spacer region, corresponding to a slight distance reduction (Fig. 2c). Whereas the measured changes in FRET efficiencies were small, we were able to accurately derive relative populations of free and ComK-bound *comG* molecules by fitting our data to superpositions of two-state Gaussian peaks (Fig. 2c, Supplementary Fig. 2, and ‘Methods’). We found that the fraction of ComK–*comG* complexes indeed increased in a sigmoidal fashion with ComK concentrations (Fig. 2d), in agreement with the cooperative mode of binding required by mathematical models of the phenotype switch^11,12,37^. Fits with the Hill equation (Fig. 2d)^38^ result in Hill exponents of *n* = 3.6 ± 0.1 for box 1 and *n* = 3.4 ± 0.3 for box 2. Independent controls confirm that ComK is monomeric in solution (Supplementary Fig. 1 and Supplementary Table 3). To interrogate coupling between box 1 and box 2, we created shorter single-box constructs (Fig. 2d and Supplementary Table 2). Remarkably, we obtained *n* = 1.7 ± 0.1 for box 1 and *n* = 1.5 ± 0.1 for box 2. Since Hill exponents provide a lower limit of the number of binding sites, these values agree with earlier reports of a 2:1 (protein:DNA) stoichiometry per box^31^ at least. The twofold higher Hill exponents in the natural promoter show that the FRET probes on one box also report on ComK binding to the other box, which requires an allosteric communication between the boxes.

### Structure of the complex

**Fig. 3:**
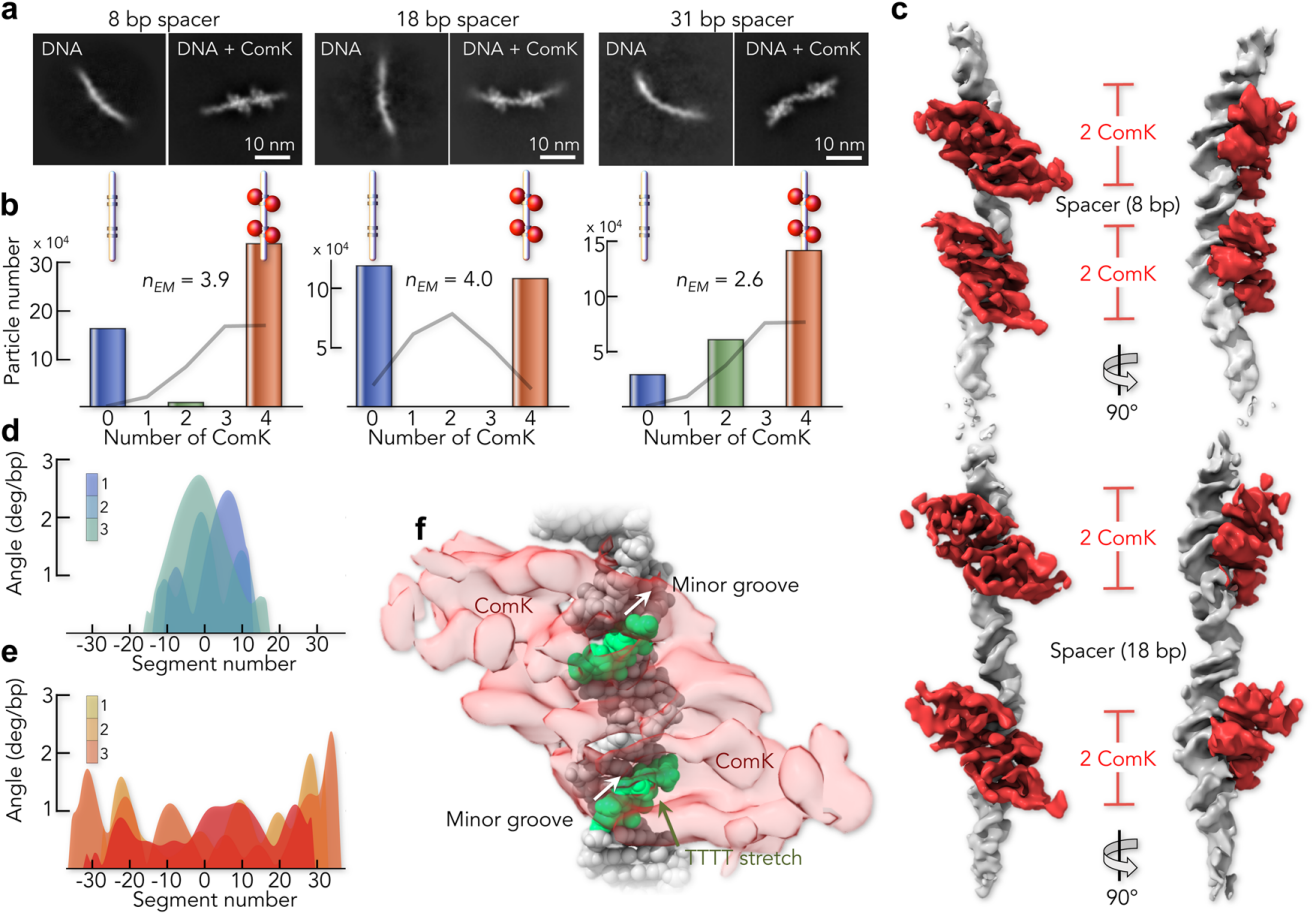
Single-particle cryo-EM analysis on ComK–DNA complexes. **a** Class averages of free DNA and the ComK–DNA complex for the promoters with an 8 bp (left), 18 bp (middle), and 31 bp (right) spacer. The scale bar is indicated. **b** Distribution of particles with different numbers of bound ComK molecules for the promoters with spacers of length 8 bp (left), 18 bp (middle), and 31 bp (right). Grey line indicates the binomial distribution for the case of no cooperativity. The Hill exponent calculated from these distributions (*n*_EM_) is indicated. **c** 3D reconstruction of ComK bound to the promoter with an 8 bp (top) and 18 bp (bottom) spacer (Supplementary Figs. 5–7). DNA (grey) and ComK (red) densities were segmented following fitting of an atomic DNA model (see ‘Methods’). **d**, **e** Curvature angles for free DNA (**d**) and the ComK–DNA complex (**e**). The 3D classes from which curvature angles were derived are shown in Supplementary Fig. 6. Colour code indicates the 3D classes. Angles between successive segments (segment length: 0.34 nm = 1 bp) for different classes were aligned with respect to the middle of the DNA. Averaged over all segments, the difference in curvature angles is 0.6°/bp between free and ComK-bound DNA. **f** Orientation of a ComK dimer (transparent red) relative to the A-tracts in a single box of the *addAB* promoter DNA (8 bp spacer). ComK faces the thymine bases (green) in the minor groove (indicated). The ComK density is shown relative to the fitted DNA model.

One classical mechanism to achieve allostery is DNA looping that allows contacts between proteins bound to different boxes^31,39^. Alternatively, ComK may bridge the boxes by interacting with the spacer, which could explain the distance decrease in the spacer upon binding of ComK (Fig. 2c, middle). To distinguish between these options, we used single-particle cryo-EM that overcomes the strong aggregation tendency and low stability of ComK (Supplementary Fig. 3). We determined 2D class averages of ComK bound to promoters with spacers of 8 bp (*addAB:* 57 bp, 35.2 kDa), 18 bp (*comG*: 93 bp, 57.5 kDa), and 31 bp (*comK*: 93 bp, 57.5 kDa). Isolated DNA complexes of the expected size were readily identified in the cryo-EM micrographs and 2D class averages revealed two major populations (Fig. 3a).

While the first population was pure promoter DNA, the second population contained DNA with extra density. Three observations are notable: (i) the extra density bound to DNA is confined to two locations that agree with the position of box 1 and box 2, thus assigning this density to ComK; (ii) the DNA in these complexes is not looped, and (iii) only a few particles with ComK density on only one box are found for promoters with 8 and 31 bp spacers (Fig. 3b). The first observation demonstrates that ComK binds specifically to box 1 and box 2, but not to the spacer between the boxes. Second, DNA looping, as suggested in previous studies^31,40^, can be excluded as an allosteric mechanism by the 2D class averages. Indeed, we did not find an increase in FRET efficiency upon ComK binding to promoters labelled at the 5′ and 3′ end (Supplementary Fig. 4). Third, the low abundance of particles with ComK on only one box even under the vitrified conditions used in cryo-EM (Fig. 3b) suggests an all-or-none binding of the protein, in accord with the high cooperativity found with smFRET at room temperature (Fig. 2d). Since Hill exponents can also be interpreted as variance of the distribution of bound ligands relative to that of the binomial distribution^41^, we computed the Hill exponents (*n*_EM_) from the cryo-EM distributions (see ‘Methods’). These values agreed qualitatively with those obtained from smFRET (Fig. 3b and Supplementary Table 4). To determine the precise arrangement of ComK on the DNA, we reconstructed the 3D maps of the complexes with spacers of 8 and 18 bp (Fig. 3c and Supplementary Figs. 5–7). To overcome the severe preferred orientation of the complexes relative to the electron beam, cryo-EM images were collected while tilting the sample (see ‘Methods’). The 3D maps show that ComK molecules in the two boxes face the same side of the DNA. In addition, we find a pseudo C_2_-symmetry of the protein density in each box, indicating two molecules of ComK per box, i.e., four per promoter, in excellent accord with the Hill exponents (Fig. 2d) and a previous report^31^. We further noted that ComK densities at individual boxes were continuous, which hints towards significant protein–protein contacts within a box (Fig. 3c). By contrast, we detected no ComK interactions across the spacer region, which suggests DNA-mediated allostery as source for the high binding cooperativity. To determine the allosteric mechanism, we analysed the DNA curvature of free (Fig. 3d) and bound promoters (Fig. 3e), and found a significant reduction in the presence of ComK, which hints at a potential mechanism. ComK is known to interact with the minor groove of DNA^31^, which is significantly narrower in A-tracts compared to B-DNA^42^. Indeed, a fit of our 3D density with an atomistic representation of the *addAB* promoter (8 bp spacer) that agrees well with our smFRET values (Supplementary Fig. 8, and Supplementary Tables 5 and 6), suggests that ComK faces the minor groove by forming contacts with the thymine bases of the A-tracts (Fig. 3f). Widening the minor groove by ComK would lower DNA curvature around a box, thus affecting the width of the minor groove at the second box. Transmitting such curvature changes from one box to another likely involves structural alterations of the spacer, which might cause the spacer contraction observed with smFRET (Fig. 2c). Importantly, the model stipulates that DNA-mediated communication between boxes ought to be dependent on the spacer length.

### Mechanical force transduction in DNA

**Fig. 4:**
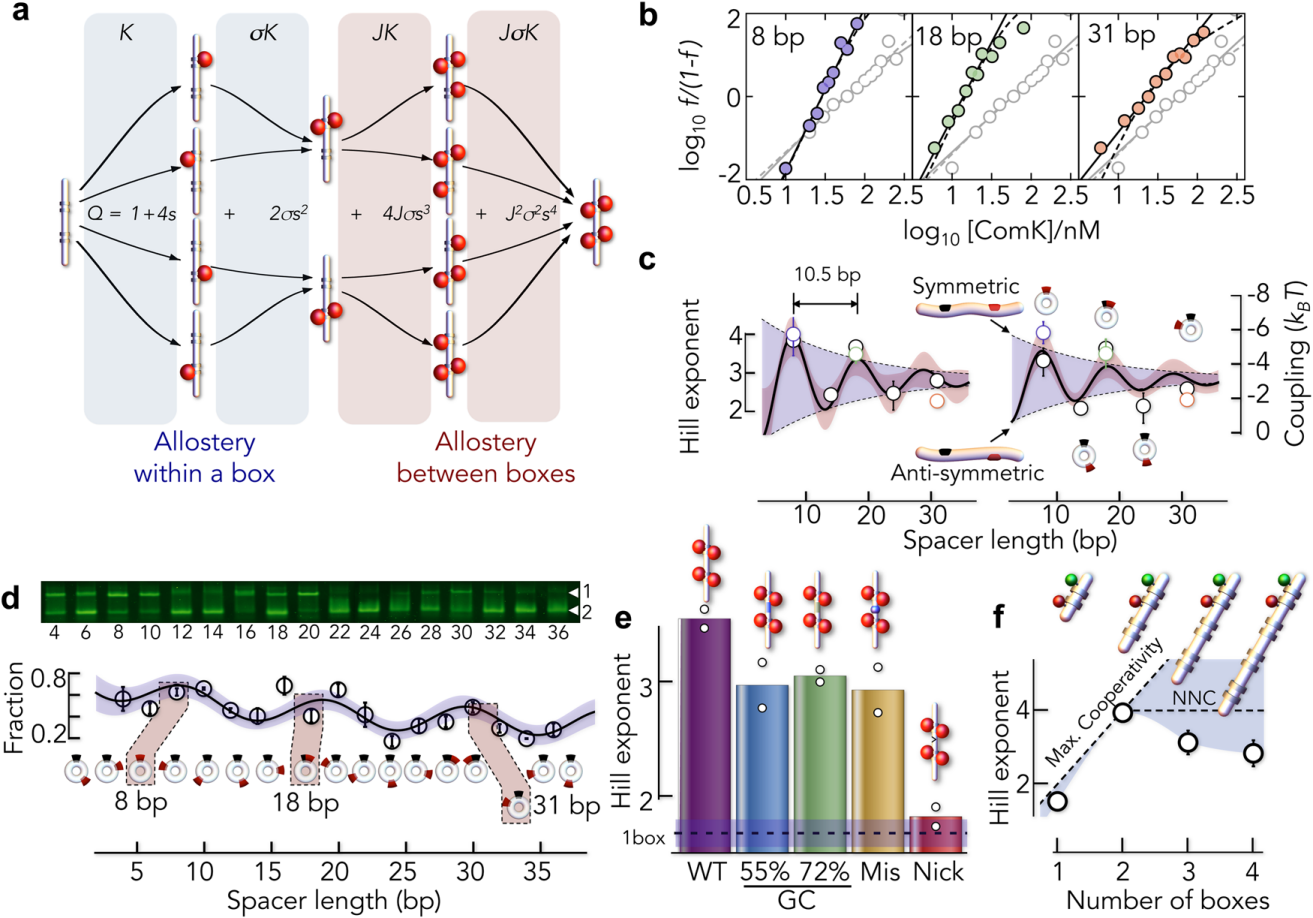
Tuning allostery. **a** Schematics of the KNF binding model (‘Methods’ Eqs. (20) and (21)) with DNA (grey) and ComK (red). A single box is first occupied with two ComK molecules before the second box is bound. The model contains a microscopic association constant *K* and the allosteric parameters *σ* and *J* due to allostery within a box and between boxes, respectively. Binding constants for each step are identical for all paths. (Middle) Binding polynomial (*Q*) of the model with *s* = *K* [ComK]. **b** Hill plots of the binding isotherms for promoters with varying spacer length (indicated) in comparison to an isolated box (grey). Solid lines are fits with the Hill equation and dashed lines are fits with the model in **a**. **c** Hill exponents (left) and inter-box coupling free energies (right) as function of the spacer length for natural promoters (coloured symbols) and for designed promoters (Supplementary Table 2). Solid black lines are fits with the elastic-coupling model (‘Methods’ Eqs. (26) and (27)). Blue shaded areas indicate the spacer-dependent damping for symmetric and antisymmetric arrangements of the boxes as indicated schematically. Red shaded areas are the 90% confidence interval of the fit. Circles represent the mean and error bars indicate the minimal and maximal values from independent measurements (*n* ≥ 2). **d** Gel assay to screen ComK binding to promoters with different spacer lengths with free DNA (lower band: 2) and ComK-bound DNA (upper band: 1) at a ComK concentration of 300 nM. (Bottom) Relative fraction of the ComK–DNA band as function of the spacer length. Promoters with 8, 18, and 31 bp are highlighted. Error bars indicate ±SD of independent triplicates. Solid line is a fit with a sum of a cosine and exponential function. Shaded area is the 90% confidence band. **e** Hill exponents for *comG* promoters with modified spacer. White circles indicate the two independent measurements. The dashed line indicates the limit of an isolated box. **f** Hill exponents of artificial promoters (8 bp spacer) with multiple boxes (circles). The diagonal line indicates maximal cooperativity and the horizontal line indicates maximum nearest-neighbour cooperativity (NNC). Error bars of the Hill exponents represent the error of the fit with Eq. (22).

To test this mechanism, we altered the spacer length and measured the effect on cooperativity using a Koshland-Nemethy-Filmer (KNF) binding model^5^ that is derived from the cryo-EM structures (Fig. 4a). Each box has two binding sites for ComK. The first ComK binds a box with the association constant *K*. The second molecule binds the same box with *σ*-fold increased affinity due to protein–protein contacts between the ComK molecules. Once the first box is saturated with ComK, the altered DNA curvature increases the affinity at the second box by a factor *J*. The quantity Δ*g*_*J*_ = −*k*_B_*T* log*J* is the DNA-mediated coupling free energy between two boxes. Using this model, we examined the coupling between boxes using the three promoters *addAB*, *comG*, and *comK* with spacer lengths of 8, 18, and 31 bp, respectively (Fig. 4b). When analysing the binding isotherms (Fig. 4b), we found a significant decrease in the Hill exponent with increasing spacer length, which is also found in the ligand distribution derived from cryo-EM (Fig. 3b). Similarly, the coupling free energy (−Δ*g*_*J*_) drops from 5.8 ± 0.9 *k*_B_*T* (8 bp) over 4.5 ± 1.2 *k*_B_*T* (18 bp) to 1.9 ± 0.1 *k*_B_*T* (31 bp). The result shows that spacer length is key for allostery, in line with the idea that reducing promoter curvature is the main origin for allosteric signal transmission. Compared to allosteric effects found previously in artificial promoters without curvature^14,23^, the coupling free energies are almost threefold larger, which converts to a 40-fold stronger effect on the association constants. Hence, changes in curvature, though not mandatory^14,23^, greatly enhance DNA-mediated allostery. An elastic-coupling theory of DNA bending predicts a decrease of Δ*g*_*J*_ with increasing spacer length^18^. Yet, this decay should be modulated by the helical periodicity of the DNA (see ‘Methods’). For instance, boxes in the natural promoters *addAB* and *comG* are one and two helical turns away from each other, respectively, thus leading to symmetric arrangements in which ComK always faces the same side of the DNA (Fig. 3c). We expect antisymmetric arrangements with non-integer turns to have significantly lower cooperativity (see ‘Methods’)^18^. We therefore designed five promoters with 8, 14, 18, 24, and 31 bp spacers (Supplementary Table 2). Indeed, we find that non-integer turns show much lower cooperativity (Fig. 4c and Supplementary Table 4), resulting in a modulation of Hill exponents and coupling free energies. To determine the modulation period more precisely, we performed a qualitative gel assay that probes the fraction of ComK–DNA complexes and shows good agreement with the B-DNA periodicity of 10.5 bp (Fig. 4d). With this value, we used elastic-coupling theory^18^ to fit our experimental Hill coefficients and coupling free energies (Fig. 4c, and see ‘Methods’ Eqs. (26) and (27)). The model predicts a dampening of the allosteric coupling with increasing spacer length. Dampening is characterized by the decay length *ξ* = (*k*_B_*Tl*_p_/*f*)^1/2^ with the persistence length *l*_p_ = 40 nm (ref. ^43^) and a tension force *f*. We found *ξ* = 14 ± 8 bp and *f* ~ 7 pN. The model also provides the average change in bending 1.1 ± 0.3°/bp (see ‘Methods’), which is in good accord with the average change determined from the cryo-EM structures (0.6°/bp; Fig. 3d, e). Yet, since bending is not uniform across the promoter, any comparison of average bending angles between cryo-EM and fits with the elastic-coupling theory remain qualitative.

### Tuning allostery via DNA sequences

To understand the sequence determinants of allostery, we altered the spacer sequence in *comG* by increasing its GC content, thus increasing DNA stability. The GC content of the native spacer (40%) is close to the genome average^44^. We generated two random spacer sequences of higher GC content (55 and 72%, Supplementary Table 2). Indeed, increasing GC content lowered cooperativity (Fig. 4e). Nevertheless, the Hill exponents remained above the values found for the isolated boxes, suggesting that spacer sequences fine-tune allostery at best. Next, we tested whether allostery requires a correct base pairing by introducing a mismatch of four bases at the centre of the spacer. However, even though the Hill exponent is reduced compared to wild-type *comG*, it is comparable to those found for increased GC contents (Fig. 4e). Remarkably, this result suggests that the allosteric communication between boxes does not stringently require a continuous base pair stacking in the spacer. These results point at the DNA backbone as major determinant for allosteric signal transmission. Indeed, when we introduce a nick in the spacer, cooperativity is abolished and we obtain a Hill exponent of *n* = 1.8 ± 0.1, i.e., close to the value found for the isolated boxes (Fig. 4e). Clearly, nicked DNA has greater flexibility and a quantitative comparison with the effects found in double-stranded DNA has to be taken with care. Yet, given that neither a higher GC content nor a mismatch show comparable effects, we conclude that curvature-induced tension between the boxes propagates mainly via an intact DNA backbone, but it can be fine-tuned by the spacer sequence and length.

Finally, we tested promoters with multiple boxes. In fact, the *comG* promoter contains an A-tract located 10 bp upstream of the first box (Supplementary Table 2)^40^ that is not occupied in our cryo-EM structure and controls indeed show that ComK is incapable of binding isolated A-tracts (Supplementary Fig. 9 and Supplementary Table 7). However, increasing the number of perfect boxes may be a simple strategy to further boost cooperativity. Yet, even the presence of four boxes with short spacing (8 bp) does not increase the Hill exponent beyond the value found for the promoter with two boxes. This implies that curvature changes do not propagate across multiple boxes, but rather mediate nearest-neighbour communication (Fig. 4f).

## Discussion

Our results show that DNA-mediated allostery generates high cooperativity in TF binding via mechanical deformations of the DNA across distances larger than 6 nm (18 bp). Notably, this mechanism is a built-in feature of natural ComK promoters to filter copy number fluctuations of ComK (Fig. 1b, c): copy numbers below the midpoint of the Hill curve leave the promoter unoccupied, those above the midpoint cause near full occupation. With increasing cooperativity, this filter increases the variability (noise) of free and bound promoters, which would also affect the ratio of vegetative and competent cells in a population. However, even if genetic circuits function in the deterministic regime of high copy numbers, sigmoidal dose responses of gene expression activity with TF concentrations are key for their dynamics^45–47^. Altering the steepness of this response, i.e., the molecular cooperativity, will unambiguously alter these dynamics^10,48^. Hence, besides the architecture of wiring genes^49,50^, designing dose–response curves via promoter sequences will provide a more precise level of engineering the dynamics of gene networks in the future.

## Methods

### DNA labelling and duplex preparation

Single-stranded DNA constructs (Supplementary Tables 1 and 2) were synthetized and HPLC-purified by the Keck Biotechnology Resource Laboratory (Yale School of Medicine). For labelling with the amino group-reactive dyes AlexaFluor 488 (donor) and AlexaFluor 594 (acceptor) NHS ester (Thermo Fisher), thymine or adenine bases at the labelling positions contained an amino modifier C6-dT/dA CE phosphoramidite (Glen Research; Supplementary Tables 1 and 2). In constructs used for smFRET ComK-binding experiments, the positions of the modified A- and T-bases were chosen such that nucleotides that had previously been shown to be protected by ComK binding^31^ were avoided. For labelling, we followed the procedure by Masoud et al.^51^ with the exception that the labelling reaction was incubated for 1 h at 45 °C. The donor and acceptor dyes were used to label the forward and reverse ssDNA strands, respectively. Reversed-phase HPLC was used to remove unreacted dye, and unlabelled ssDNA. To this end, a ZORBAX Eclipse Plus C18 (3.5 μm) column (Agilent) was used, equilibrated with TEAA buffer (0.1 M trimethylamine buffered with acetic acid glacial to pH 6.5 and supplemented with 5% acetonitrile). A gradient of 7–30% acetonitrile over 40 ml was used for the separation. Labelled fractions were dried overnight (SpeedVac) and dissolved in 50 μl ddH_2_O. DNA and dye concentrations were quantified by UV–VIS photometry, using the extinction coefficients of the free dyes *Ɛ*_495_ = 73,000 M^−1^ cm^−1^ (AlexaFluor 488) and *Ɛ*_590_ = 92,000 M^−1^ cm^−1^ (AlexaFluor 594). Only fractions containing 1:1 ratio of dye:ssDNA were used for smFRET experiments. Annealing of ssDNA donor and acceptor strands for smFRET experiments was obtained by mixing 2 pmol donor-labelled ssDNA with 3 pmol acceptor-labelled ssDNA in 50 mM Tris pH 7.5, 0.1 M NaCl, and 3 mM Mg_2_Cl. The samples were heated to 95 °C and cooled gradually over 1 h in a PCR cycler. Unlabelled DNA duplexes for cryo-EM and EMSAs (electrophoretic mobility shift assays) were obtained by mixing a 1:1 ratio of forward and reverse ssDNA strands, using the same annealing procedure.

### Expression and purification of ComK

The sequence of ComK (Uniprot ID: P40396), codon-optimized for expression in *Escherichia coli* (Genscript; Supplementary Table 3), was inserted into the NcoI and HindIII sites of a pET28b vector, and the protein was expressed in inclusion bodies (IBs) in BL21(DE3). Bacteria were grown in 4 l LB medium containing 50 μg/ml kanamycin. Cultures were induced at OD_600_ 0.7 with 0.3 mM IPTG. After 4 h at 37 °C, the cells were harvested by centrifugation and the cell pellets were re-suspended in lysis buffer (50 mM Tris pH 8.0, 10 mM EDTA, 100 μM PMSF, 1.2 μg/ml leupeptin, and 1 μM pepstatin A). After sonication (70% power, pulse on 2 s, pulse off 10 s, 60 cycles, on ice) and centrifugation (48,200 × *g*, 30 min at 4 °C), the IB precipitate was mixed with 15 ml of 60 mM EDTA, 6% Triton, 1.5 M NaCl, homogenized, and incubated at 4 °C for 1 h to solubilize membrane debris. After centrifugation (48,200 × *g*, 30 min at 4 °C), the resulting IB precipitate was supplemented with 0.1 M Tris pH 8, 1 mM EDTA, homogenized, and centrifuged using the same conditions. Finally, the IB-pellet was re-solubilized (>2 h, 4 °C) in 50 mM Tris pH 8.0, 6 M GdmCl, 10 mM imidazole, containing 10 mM DTT. After complete resolubilization, the buffer was exchanged to 50 mM Tris pH 8.0, 6 M GdmCl, and 10 mM imidazole, using a HiPrep desalting column (26/10, GE) to remove DTT. The protein containing fractions were then loaded on a HisTrap Ni-NTA column (5 ml, GE) and the protein was eluted with 50 mM Tris pH 8.0, 6 M GdmCl, and 0.5 M imidazole, using a linear gradient of 3 ml/min for 10 min. The eluted protein (20 ml) was supplemented with 0.1 mg/ml HRV3C and 5 mM DTT and the solution was dialysed overnight (4 °C) against 300 volumes of cleavage buffer (50 mM Tris and 100 mM l-Arg at pH 7, 1 mM DTT). HRV3C protease refolds efficiently under these conditions. Due to the strong aggregation propensity of ComK, the protein partially precipitates during this procedure. The protein was fully precipitated by the addition of an equivalent volume of a saturated ammonium sulfate solution. The precipitate was collected by centrifugation (48,200 × *g*, for 20 min at 4 °C) and the protein pellet was dissolved in 50 mM Tris pH 8.0, 6 M GdmCl, and 10 mM imidazole. The cleaved His-tag and HRV3C protease were removed using Ni-NTA affinity chromatography (5 ml HisTrap column, GE). Cleaved, i.e., untagged protein was obtained in the flow-through fractions, while the purification tags and HRV3C protease were retained on the column. Finally, the buffer of the ComK-containing fractions was exchanged to 0.5 M l-Arg pH 7.3 using a HiTrap desalting column (5 ml, GE). The protein concentration was determined using an extinction coefficient of *Ɛ*_280_ = 24,660 M^−1^ cm^−1^ computed from the amino acid sequence of ComK. Since ComK contains four cysteine residues, we added 10 mM DTT and stored the samples at −80 °C. For mass determination, the purified ComK in 0.5 M l-Arg was injected into a combined LC–MS system (Waters ACUITY UPLC class H) equipped with a C4 column (300 Å, 1.7 μm, 21 mm × 100 mm) and the mass was determined in positive ion mode using electrospray ionization using a desolvation temperature of 500 °C, with flow rate of 1000 l/h. The voltage was 0.69 kV for the capillary and 46 V for the cone. Spectra, de-convoluted with the MaxEnt1 software, resulted in a single peak with a mass of 22,652 ± 10 Da, in good agreement with a calculated mass of 22,640 Da.

### Labelling of ComK for 2fFCS experiments

Singly labelled ComK was used to determine the oligomeric state of ComK in solution using two-focus fluorescence correlation spectoscopy (2fFCS)^52^. Since ComK contains four intrinsic cysteine residues at positions 31, 39, 55, and 102 (Supplementary Table 3), our approach was to replace them by alanine. However, the resulting ΔCys variant was incapable of binding DNA at protein concentrations ≥100 nM as demonstrated in smFRET experiments using doubly labelled *comG* (Supplementary Fig. 1d). The result surprises given the facts (i) that ComK is a cytosolic protein that acts in a reducing environment and (ii) that all our smFRET experiments with wild-type ComK are performed under strongly reducing conditions (20 mM DTT). The result shows that the reduced cysteine residues are either crucial for the structural integrity of ComK, e.g., as metal binding centres, and/or are key for the interaction with the DNA.

To obtain active and fluorescently labelled ComK for 2fFCS measurements, we therefore introduced an additional well-accessible cysteine at the N-terminus of ComK (N-Cys ComK) for labelling. We checked that the N-terminal cysteine is labelled preferentially (Supplementary Fig. 1a inset). To this end, wild-type ComK and the variant N-Cys ComK were diluted to 10 μM and 0.9 mol equivalents of AlexaFluor 488 C5 maleimide were added to the solution. Samples (50 μl) were drawn after different times and quenched by the addition of 100 mM DTT. The samples were subjected to C18-reversed-phase chromatography and the labelling ratio of the protein elution was determined from the absorbance at 280 and 493 nm. For 2fFCS sample preparation, we added 1.4 mol equivalents of AlexaFluor 488 C5 maleimide to 46 μM ComK in 0.5 M arginine–HCl, pH 7.3. After 30 min at room temperature, the reaction was quenched by the addition of 10 mM DTT and the buffer was exchanged to 50 mM Tris-HCl pH 8.0, and 6 M GdmCl. Unlabelled ComK was separated from labelled ComK using reversed-phase chromatography (RP-C18 column) in 0.1% TFA in ddH_2_O. The protein was eluted using a gradient of 25–50% acetonitrile at a flow rate of 1 ml/min for 40 min. The fraction containing labelled ComK was first lyophilized and then dissolved in 50 mM Tris-HCl pH 8.0, and 6 M guanidinium chloride (GdmCl) buffer. A UV–Vis quantification confirmed a 1:1 ratio of ComK to dye.

### Determination of the dimerization state without labelling ComK

To obtain a labelling-independent confirmation that ComK is monomeric in solution, we performed quantitative size-exclusion chromatography experiments. Here, the exclusion volume of a Superdex 75 column (GE Healthcare) was first determined using dextran-blue and the total volume was determined by ascorbic acid. In a second step, we performed a calibration of the column using standard proteins (LMW, GE Healthcare) of known size in 20 mM Tris pH 7.0, 50 mM l-Arg, 5 mM MgCl_2_, 150 mM KCl, 20 mM DTT, and 0.001% Tween-20 (Supplementary Fig. 1c). Finally, we injected 3 and 4 μM ComK. The elution volume of ComK was in accord with a mass of 20.3 ± 0.8 kDa, indicating that ComK is a monomer at these concentrations^53^.

### Optimizing solution conditions to prevent ComK aggregation

Our results show that ComK aggregates at concentrations as low as 2 μM (Supplementary Fig. 3c, d). High concentrations of l-Arg (0.5 M) effectively prevent this aggregation up to a concentration of >100 μM. However, our experiments also show that such high l-Arg concentrations suppress the binding of ComK to DNA. A quantification of the aggregation of ComK using UV/VIS photometry revealed that at least 50 mM l-Arg (Supplementary Fig. 3) is required to ensure soluble ComK at a concentration of 2 μM. Even though the l-Arg concentration exceeds the cytosol concentration of 0.57 mM (*E. coli*)^54^, our control experiments show that the conformation of the DNA (*comG*) is unaffected by l-Arg up to a concentration of 100 mM (Supplementary Fig. 3d inset), and ComK is fully active in binding DNA (Supplementary Fig. 2b–m). We therefore used 50 mM l-Arg in all smFRET and cryo-EM experiments. Notably, l-Arg is known to stabilize proteins, thus preventing aggregation^55^; however, it does not solubilize pre-formed aggregates in our hands.

### SmFRET and 2fFCS experiments

All smFRET and 2fFCS experiments with ComK were performed at 20 mM Tris pH 7.0, 50 mM l-Arg, 5 mM MgCl_2_, 150 mM KCl, 20 mM DTT, and 0.001% Tween-20. In contrast, the smFRET experiments, in which we map the curvature of the *comG* promoter (Fig. 2a, b) were performed in 20 mM sodium phosphate pH 7 or 4, 100 mM β-mercapto-ethanol, and 0.001% Tween-20, which allowed an easier adjustment of the solution pH. The ionic strength was kept constant at 100 mM in these experiments by the addition of NaCl. The concentration of labelled DNA was 30 pM.

All smFRET experiments were performed with a MicroTime 200 confocal microscope (PicoQuant) equipped with an Olympus IX73 inverted microscope. Briefly, we used linearly polarized light from a 485 nm diode laser (LDH-D-C-485, PicoQuant) and unpolarized light at 594 nm from a supercontinuum light source (Solea, PicoQuant) to excite donor and acceptor alternatingly with a repetition rate of 20 MHz in an interleaved manner, thus resulting in a period length of 50 ns that contains one donor and one acceptor pulse. This pulsed-interleaved excitation (PIE) scheme^56^ was used to distinguish molecules with only donor dye from those carrying both dyes, but with a large inter-dye distance (Supplementary Fig. 2a). The excitation beam was guided through a major dichroic mirror (ZT 470-491/594 rpc, Chroma) to a 60×, 1.2NA water objective (Olympus) that focuses the beam into the sample. The sample was placed in a home-made cuvette with a volume of 50 μl made of round quartz cover slips of 25 mm diameter (Esco Optics) and borosilicate glass 6 mm diameter cloning cylinder (Hilgenberg), using Norland 61 optical adhesive (Thorlabs). All measurements were performed at a laser power of 100 μW (485 nm) and 20 μW (594 nm) measured at the back aperture of the objective. Photons emitted from the sample were collected by the same objective and after passing the major dichroic mirror (ZT 470-491/594 rpc, Chroma), the residual excitation light was filtered by a long-pass filter (BLP01-488R, Semrock) and focused on a 100 µm pinhole. The sample fluorescence was detected either with two channels (Fig. 2a, b) or four channels (all other smFRET experiments). Donor and acceptor fluorescence was separated via a dichroic mirror (T585 LPXR, Chroma), and each colour was focused onto a single-photon avalanche diode (SPAD; Excelitas) with additional bandpass filters: FF03-525/50, (Semrock) for the donor SPAD and FF02-650/100 (Semrock) for the acceptor SPAD. The arrival time of every detected photon was recorded with a HydraHarp 400 M time-correlated single-photon counting module (PicoQuant) and stored with a resolution of 32 ps.

For 2fFCS^52^, the sample was excited with two orthogonally polarized lasers at 485 nm (LDH-D-C-485, PicoQuant). The beams of both lasers were combined and laterally shifted by a Nomarsky prism at the back aperture of the objective. Emitted light passed through a 150 μm pinhole. Afterwards, the emitted light was split using a polarizing beam splitter and focused on two separate SPAD detectors. The distance between the two resulting foci was experimentally determined using the known Stokes radii (*R*_S_) of the calibration samples: the dye Oregon Green (*R*_S_ = 0.6 nm, 0.36 kDa) and the proteins aprotinin (*R*_S_ = 1.29 nm, 6.5 kDa), RNaseA (*R*_S_ = 1.73 nm, 13.7 kDa), carboxy anhydrase (*R*_S_ = 2.19 nm, 29 kDa), ovalbumin (*R*_S_ = 2.65 nm, 44 kDa), and conalbumin (*R*_S_ = 3.1 nm, 75 kDa; Supplementary Fig. 1a, b). For calibration, all proteins were labelled with AlexaFluor 488 NHS ester at primary amino groups of lysine residues. The autocorrelation functions of the signal of each focus and the two cross-correlation functions were fit globally with a model that contained one diffusion component and two exponential decay components to account for the triplet dynamics of the dyes (Supplementary Fig. 1a). The global fits were performed iteratively to minimize the squared difference between the calibration values and the values determined with 2fFCS. Subsequently, the Stokes radius of labelled ComK was determined in the absence and presence of increasing amounts of unlabelled ComK (Supplementary Figs. 1b and 3a). All measurements were performed at 23 °C.

### Determination of the thermodynamic stability of ComK

To understand the strong aggregation tendency of ComK, we used 2fFCS to determine the stability of ComK under physiological conditions. To this end, we denatured labelled ComK at different concentration of the denaturant GdmCl. With increasing concentrations of GdmCl, the Stokes radius increases in a cooperative manner (Supplementary Fig. 3b). To obtain the stability, we fit the data using a two-state model of protein folding. Assuming a spherical shape of folded and unfolded ComK, the average Stokes radius $\left\langle R\right\rangle$ at any concentration of GdmCl is given by

1 $$\left\langle R\right\rangle =\root3\of{\frac{3}{4\pi }\left[f\,{v}_{f}+\left(1-f\right){v}_{u}\right]}$$

here, *f* is the fraction of folded molecules, *v*_*f*_ and *v*_*u*_ are the volumes of folded and unfolded molecules, respectively. Since it is known that the dimension of unfolded polypeptide chains responds sensitively to the concentration of denaturants^57–59^, we assume that the volume of the unfolded state changes linearly with the concentration of GdmCl (*x*) according to

2 $${v}_{u}={m}_{u}x+{n}_{u}$$

with the slope *m*_*u*_ and the intercept *n*_*u*_. The fraction *f* for a two-state system is given by

3 $$f={e}^{-\Delta G/RT}/(1+{e}^{-\Delta G/RT})$$

here, *R* is the ideal gas constant, *T* is the temperature in Kelvin, and Δ*G* is the free energy difference between folded and unfolded molecules. We further assume a linear free energy relationship of the type

4 $$\Delta G={mx}+\Delta {G}_{0}$$

The data in Supplementary Fig. 3b were fit using a combination of Eqs. (1)–(4). The fit results in free energy of stabilization of Δ*G*_0_ = −8.8 ± 0.3 kJ/mol.

### SmFRET data analysis

Instrumental imperfections and differences in the brightness of donor and acceptor require a correction of the detected raw photon counts in the two channels (1 and 2)^60,61^. These corrections are defined by five parameters: *γ*_*1*_ and *γ*_*2*_ that account for the different detection probabilities of photons from the two dyes, *β*_21_ and *β*_12_, the leakage of donor photons into the acceptor channel 1 and the leakage of acceptor photons into the donor channel 2, respectively, and *α*, the probability to directly excite the acceptor dye at the wavelength specific for the donor. If *n*_*1*_ and *n*_*2*_ are the detected photons in the acceptor and donor channel, respectively, and *b*_*1*_ and *b*_*2*_ are the background rates in both channels, the corrected photon counts for acceptor and donor (*n*_A_′ and *n*_D_′) are given by

5 $$\left(\begin{array}{c}{n}_{A}\\ {n^{\prime}}_{D}\end{array}\right)=\left(\begin{array}{cc}{\gamma }_{1} & -{\beta }_{DA} \\ {-\beta }_{AD} & {\gamma }_{2}\end{array}\right)\left(\begin{array}{c}{n}_{1}-{b}_{1}T\\ {n}_{2}-{b}_{2}T\end{array}\right){\mathrm{and}}\;{n}_{A}^{\prime }={n}_{A}-\alpha \big({n}_{D}^{\prime }+{n}_{A}\big)$$

here, *T* is a time specifying the length of a burst. The correction parameters were determined^62^ with two separate samples of the dyes, in which their concentrations were adjusted such that both samples have an absorbance of 0.1 at the excitation wavelength of 485 nm. Setting arbitrarily *γ*_1_ = 1, we obtain *γ*_2_ = 1.12 ± 0.09, *β*_DA_ = 0.050 ± 0.003, and *β*_AD_ = 0.0021 ± 0.0004 over 5 years with 21 measurements of these correction factors. We note that using these correction factors for different DNA samples assumes that the fluorescence quantum yields of donor and acceptor do not depend on the specific labelling position on the DNA. The probability of directly exciting the acceptor dye at 485 nm is given by $\alpha ={\epsilon }_{\mathrm{A}}/\left({\epsilon }_{\mathrm{A}}+{\epsilon }_{\mathrm{D}}\right)$, where ${\epsilon }_{\mathrm{A}}$ and ${\epsilon }_{\mathrm{D}}$ are the extinction coefficients of the dyes at the donor excitation wavelength of 485 nm. For our dye pair, *α* = 0.049. Transfer efficiency histograms were computed with the fully corrected photon counts according to

6 $$E={n}_{\mathrm{A}}^{{\prime} }/\left({n}_{\mathrm{A}}^{{\prime} }+{n}_{\mathrm{D}}^{{\prime} }\right)$$

Importantly, the corrections (Eq. (5)) are already taken into account during burst identification. Bursts were identified from the measured photon traces following Eggeling et al.^60^ and Hoffmann et al.^61^. If $\Delta {t}_{i}={t}_{i}-{t}_{i-1}$ is the inter-photon time of the *i*th photon, the photon is retained if $\Delta {t}_{i}\le {\gamma }_{j}\left(i\right)\Delta {t}_{{\max }}$ ($\Delta {t}_{{\max }}=100\,\upmu {\mathrm{s}}$) with ${\gamma }_{j}\left(i\right)$ being the correction factor of the *i*th photon detected in channel $j=\left[{\mathrm{1,2}}\right]$ (Eq. (5)). The algorithm then proceeds to the next photon *i* + 1, stops after *n* photons once $\Delta {t}_{i+n} \,> \, {\gamma }_{i}\left(i+n\right)\Delta {t}_{{\max }}$, and provides the total length of the burst by $T={t}_{n-1}-{t}_{i-1}$. The resulting string of photons is now corrected via Eq. (5), using estimated background rates *b*_1_ and *b*_2_. The initial guess of *b*_1_ and *b*_2_ is given by all detected photons in channels 1 and 2, respectively, divided by the total measurement time. A burst is identified if ${n^{\prime} }_{\mathrm{A}}+{n^{\prime} }_{\mathrm{D}} \,> \, 80$. The photons belonging to this burst are removed from the photon trace, and a new guess for *b*_1_ and *b*_2_ is computed based on the remaining photons. Subsequently, the burst search is performed again with updated background rates. This procedure converges after three iterations to constant background rates and a constant number of identified bursts. Since the identified bursts also contain molecules for which the acceptor bleached during the transit trough the confocal spot, thus masking the true transfer efficiency, we further cleaned the FRET histograms by these events^63^. For a burst with *n*′_D_ donor photons with the arrival times ${t}_{{\mathrm{D}},1}{\ldots} {t}_{\mathrm{D}},{n^{\prime} }_{{\mathrm{D}}}$ and *n*′_A_ acceptor photons with the arrival times ${t}_{{\mathrm{A}},1}{\ldots} {t}_{\mathrm{A}},{n^{\prime} }_{{\mathrm{A}}}$, the average arrival times are given by $\left\langle {t}_{\mathrm{D}}\right\rangle ={n^{\prime} }_{\mathrm{D}}^{-1}{\sum}_{i}{t}_{{\mathrm{D}},i}$ and $\left\langle {t}_{\mathrm{A}}\right\rangle ={n}_{\mathrm{A}}^{{\prime} -1}{\sum}_{i}{t}_{{\mathrm{A}},i}$. The burst asymmetry is defined by ${\alpha }_{{\mathrm{DA}}}=\left\langle {t}_{\mathrm{D}}\right\rangle -\left\langle {t}_{\mathrm{A}}\right\rangle$. If the acceptor dye bleaches, we find ${\alpha }_{{\mathrm{DA}}} \,> \, 0$. Taking shot noise into account, the distribution of ${\alpha }_{{\mathrm{DA}}}$ has a standard deviation given by

7 $${\sigma }_{{\mathrm{DA}}}=\frac{T}{2\sqrt{3}}{\left(\frac{1}{{n^{\prime} }_{\mathrm{D}}}+\frac{1}{{n^{\prime} }_{\mathrm{A}}}\right)}^{1/2}$$

To eliminate molecules with a bleached acceptor, we excluded all molecules for which ${\left|{\alpha }_{{\mathrm{DA}}}\right| > \sigma }_{{\mathrm{DA}}}$.

In addition, only molecules containing active acceptor and donor dyes were included in the analysis (Supplementary Fig. 2a). To this end, we computed the donor–acceptor stoichiometry (*S*) for each burst according to

8 $$S=\frac{{n}_{{\mathrm{DD}}}^{{\prime} }+{n}_{{\mathrm{DA}}}^{{\prime} }}{{n}_{{\mathrm{DD}}}^{{\prime} }+{n}_{{\mathrm{DA}}}^{{\prime} }+{\gamma }_{{\mathrm{PIE}}}{n}_{{\mathrm{AA}}}}$$

here, *γ*_PIE_ ∼ 2.5 is a correction factor to account for the different excitation intensities for donor and acceptor. Furthermore, the first subscript indicates the emission and the second subscript indicates the excitation. Only molecules with *S* < 0.8 were used for constructing smFRET histograms.

### Determination of the fraction of ComK-bound promoters from smFRET

FRET histograms for ComK-binding experiments were fitted with a combination of two Gaussian distributions^64^, since all constructs used in these experiments had FRET values close to 0.5, thus showing no skewing. The width and position of these FRET peaks were fixed to the values obtained for the FRET distribution in the absence of ComK and in the presence of saturating amounts of ComK, which minimized the number of free fitting parameters. Only the amplitudes of the Gaussian peaks were free fitting parameters. The area under the histogram curve for each subpopulation was determined using numerical integration to obtain the fraction of ComK-bound promoter.

FRET histograms of the DNA constructs used to identify curvature in the *comG* promoter (Fig. 2a, b and Supplementary Fig. 2a) were fitted with Gaussian distributions for constructs with intermediate FRET efficiency, and with log-normal functions for histograms with mean FRET values close to 0 and 1.

### Fluorescence lifetime analysis

The average lifetimes of the donor in the presence of acceptor *τ*_DA_ (Fig. 2a, b inset) were computed from the mean arrival time of the photons relative to the laser pulse exciting the donor. The expected value of the fluorescence lifetime of the donor in the presence of the acceptor ${\tau }_{{\mathrm{DA}}}$ for a static inter-dye distance (dashed line in Fig. 2a, b insets) is given by ${\tau }_{{\mathrm{DA}}}={\tau }_{\mathrm{D}}\left(1-E\right)$, where ${\tau }_{\mathrm{D}}$ = 3.6–4 ns is the lifetime of the donor in the absence of acceptor as determined from molecules lacking an active acceptor. However, if the *comG* promoter would fluctuate between different conformations, thus sampling a distance distribution $P\left(r\right)$, the simple linear relationship between the average donor fluorescence lifetime $\left\langle {\tau }_{{\mathrm{DA}}}\right\rangle$ and the average FRET efficiency $\left\langle E\right\rangle$ will deviate from the linear Förster-dependence for a static distance^65,66^. The resulting deviation can be used to determine the width of the distribution $P\left(r\right)$. We therefore fit the peak position of the average fluorescence lifetime $\left\langle {\tau }_{{\mathrm{DA}}}\right\rangle$ and mean FRET efficiency $\left\langle E\right\rangle$, using a Gaussian distance distribution with mean $\mu$ and width $\sigma$. The analytical expressions for the average lifetime and FRET efficiency are given by

9 $$\langle {\tau }_{DA}\rangle ={\int }_{0}^{{\rm{\infty }}}{{\tau }_{DA}\left(r\right)}^{2}P\left(r\right){dr}/{\int }_{0}^{{\rm{\infty }}}{\tau }_{DA}\left(r\right)P\left(r\right){dr}$$

and

10 $$\left\langle E\right\rangle ={\int }_{0}^{{\rm{\infty }}}E\left(r\right)P\left(r\right){dr}/{\int }_{0}^{{\rm{\infty }}}P\left(r\right){dr}$$

here, ${\tau }_{{\mathrm{DA}}}\left(r\right)={\tau }_{\mathrm{D}}{\left[1+{\left({R}_{0}/r\right)}^{6}\right]}^{-1}$ and $E\left(r\right)={R}_{0}^{6}/\left({R}_{0}^{6}+{r}^{6}\right)$. The dependence between $\left\langle {\tau }_{{\mathrm{DA}}}\right\rangle$and $\left\langle E\right\rangle$ is implicit and depends on $\mu$, which is a variable, and $\sigma$, which is the fitting parameter. We obtained $\sigma$ = 0.76 nm at pH 7 and $\sigma$ = 0.66 nm at pH 4. Assuming spherically symmetric and Gaussian distributed dye clouds for donor and acceptor due to the long C_6_-alkyl linkers of our dyes, we can compute the distribution width ${\sigma }_{{\mathrm{dye}}}$ of these clouds. Assuming that donor and acceptor have the same spatial distribution (same ${\sigma }_{{\mathrm{dye}}}$), we find ${\sigma }_{{\mathrm{dye}}}=\sigma /\sqrt{2}$, which results in ${\sigma }_{{\mathrm{dye}}}$ = 0.54 nm at pH 7 and ${\sigma }_{{\mathrm{dye}}}$ = 0.47 nm. These widths are shown as radius of the spheres in the schemes in Fig. 2a, b (inset) and are very similar to the positional uncertainty (*L*) of the dye positions found previously by Wozniak et al.^34^ (see Eq. (11) below).

### Calculation of the FRET values for B-DNA

To determine the transfer efficiency profile of B-DNA (Fig. 2b, c), we followed the work of Clegg et al.^67^ and Wozniak et al.^34^ Keeping the nomenclature of Wozniak et al., the mean distances *R*_DA_ of donor (D)–acceptor (A) pairs on B-DNA is given by

11 $${R}_{{\mathrm{DA}}}=\sqrt{{\left(L+{\Delta }_{{\mathrm{bp}}}{z}_{{\mathrm{bp}}}\right)}^{2}+{r}_{\mathrm{A}}^{2}+{r}_{\mathrm{D}}^{2}-2{r}_{\mathrm{A}}{r}_{\mathrm{D}}{\cos }\left(\alpha +{\Delta }_{{\mathrm{bp}}}{\beta }_{{\mathrm{bp}}}\right)}$$

here, *α* is the offset angle between the fluorophores if they were both bound to the same base. *L* accounts for the fact that projections of the centres of D and A onto the helix axis do not necessarily coincide with the position of bases. The values *r*_A_ and *r*_D_ are the distances from the helical axis due to the dye linkers, z_bp_ is the increase along the axis per base pair, and *β*_bp_ is the increase in D–A angle per base pair. For dyes with a similar size of those used here, Wozniak et al. found *L* = 0.617 nm, *z*_bp_ = 0.338 nm, *r*_A_ = 1.177 nm, *r*_D_ = 1.247 nm, *β*_bp_ = 36°, and *α* = 89.8° for B-DNA, and we used the same parameters to compute the transfer efficiency via

12 $${E}_{{\mathrm{mp}}}={R}_{0}^{6}/\left({R}_{0}^{6}+{R}_{{\mathrm{DA}}}^{6}\right)$$

with *R*_0_ = 5.4 nm^57^. Since our experimentally measured transfer efficiencies are an average over the positional distribution of the dyes due to the flexibility of their linkers, the ${E}_{{mp}}$ values were converted to the mean transfer efficiencies $\left\langle {E}\right\rangle$ accessible in our experiments by solving the empirical implicit equation^34^

13 $${E}_{{\mathrm{mp}}}=0.008+0.679\left\langle E\right\rangle +1.470\left\langle E\right\rangle ^{2}-1.141\left\langle E\right\rangle ^{3}$$

These values are shown as black line in Fig. 2a, b. Notably, assuming that the *comG* promoter is in a B-DNA conformation at pH 4, we also used Eqs. (11)–(13) in a fit with ${R}_{0}$ as a free parameter and obtained a value of 5.6 ± 0.1 nm, i.e., very similar to the literature value of 5.4 nm used in Fig. 2a, b. Yet, this comparison between experimental smFRET values and the described model is based on two assumptions, (i) distance fluctuations of the DNA are marginal and (ii) dye motions caused by the flexible linkers are slower than the FRET transfer rate. Since we cannot unambiguously exclude violations of these assumptions, the described agreement has to be considered qualitative.

### Thermodynamic ComK–DNA-binding model

To determine coupling free energies between the binding boxes of ComK, we fit the data using a mechanistic binding model (Fig. 4a). In this model, we denote the promoter as $P$ and the four binding sites are denoted by subscripts ${P}_{1}{\ldots} {P}_{4}$. Here, the first two binding sites are in box 1 and the second two binding sites are in box 2. We further denote ComK as $X$ such that the concentration of the promoter with one ComK bound to the first site is $\left[{P}_{1}X\right]$. The concentration of free ComK is $x$. The initial binding of a ComK to each site is given by four association reactions with identical association constant *K* defined by

14 $$K=\frac{\left[{P}_{1}X\right]}{\left[P\right]x}=\frac{\left[{P}_{2}X\right]}{\left[P\right]x}=\frac{\left[{P}_{3}X\right]}{\left[P\right]x}=\frac{\left[{P}_{4}X\right]}{\left[P\right]x}$$

The second ComK binds exclusively to a box that already contains a bound ComK. We introduced this constraint based on the determined cryo-EM structures that indicates substantial protein–protein contacts between ComK molecules in one box (Fig. 3c). Hence, binding the second ComK gives the relations

15 $$\sigma K=\frac{\left[{P}_{12}{X}_{2}\right]}{\left[{P}_{1}X\right]x}=\frac{\left[{P}_{12}{X}_{2}\right]}{\left[{P}_{2}X\right]x}=\frac{\left[{P}_{34}{X}_{2}\right]}{\left[{P}_{3}X\right]x}=\frac{\left[{P}_{34}{X}_{2}\right]}{\left[{P}_{4}X\right]x}$$

The factor *σ* accounts for the higher affinity of binding a second ComK to a box due to allostery via protein–protein contacts. The third ComK now binds to a different box, thus leading to

16 $${JK}=\frac{\left[{P}_{134}{X}_{3}\right]}{\left[{P}_{34}{X}_{2}\right]x}=\frac{\left[{P}_{234}{X}_{3}\right]}{\left[{P}_{34}{X}_{2}\right]x}=\frac{\left[{P}_{123}{X}_{3}\right]}{\left[{P}_{12}{X}_{2}\right]x}=\frac{\left[{P}_{124}{X}_{3}\right]}{\left[{P}_{12}{X}_{2}\right]x}$$

where the factor *J* accounts for the inter-box cooperativity. Finally, the last ComK molecule binds to the remaining site that now includes intra-(*σ*) and inter-(*J*) box cooperativity

17 $$J\sigma K=\frac{\left[{P}_{1234}{X}_{4}\right]}{\left[{P}_{234}{X}_{3}\right]x}=\frac{\left[{P}_{1234}{X}_{4}\right]}{\left[{P}_{134}{X}_{3}\right]x}=\frac{\left[{P}_{1234}{X}_{4}\right]}{\left[{P}_{124}{X}_{3}\right]x}=\frac{\left[{P}_{1234}{X}_{4}\right]}{\left[{P}_{132}{X}_{3}\right]x}$$

Replacing $s={Kx}$ for clarity and with $\left[{P}_{0}\right]$ as the total concentration of promoter DNA, we obtain the mass conservation equation

18 $$\left[{P}_{0}\right]=\left[P\right]\left(1+4s+2\sigma {s}^{2}+4J\sigma {s}^{3}+{J}^{2}{\sigma }^{2}{s}^{4}\right)$$

which gives the binding polynomial shown in Fig. 4a

19 $$Q=1+4s+2\sigma {s}^{2}+4J\sigma {s}^{3}+{J}^{2}{\sigma }^{2}{s}^{4}$$

The fraction of fully bound promoter is therefore given by

20 $$f=\frac{{J}^{2}{\sigma }^{2}{s}^{4}}{1+4s+2\sigma {s}^{2}+4J\sigma {s}^{3}+{J}^{2}{\sigma }^{2}{s}^{4}}$$

With the same logic, we obtain for the isolated boxes

21 $$f=\frac{{\sigma s}^{2}}{1+2s+\sigma {s}^{2}}$$

Notably, these equations only hold under conditions at which the concentration of DNA is very low compared to ${K}^{-1}$, which, with $\left[{P}_{0}\right]$ = 30 pM is given at all our experimental conditions. To analyse our binding data, we first fit the data of the isolated boxes 1 and 2 globally, using Eq. (21) (Fig. 2d). Here, the value $\sigma ={e}^{-\Delta {g}_{\sigma }}$ was a global parameter, whereas $K={e}^{-\Delta {g}_{K}}$ was a local parameter that differed between box 1 and box 2. These fits provide the free energy changes Δ*g*_*σ*_ and Δ*g*_*K*_ in Supplementary Table 4. To reduce the number of parameters in Eq. (20), we kept the value of Δ*g*_*σ*_ in all fits of promoters with two boxes and expressed the coupling between two boxes as ${J}={{e}}^{-\Delta {{g}}_{{J}}}$ with the coupling free energy Δ*g*_*J*_ (Supplementary Table 4). For promoters in which we mapped both boxes (*addAB* and *comG*), we fit the data using a global value for Δ*g*_*J*_ (Figs. 2e and 4b). To allow an easier comparison of the cooperativity in the different constructs, all data were also fitted using the standard Hill equation

22 $$f={x}^{n}/\left({K}_{\text{Hill}}^{n}+{x}^{n}\right)$$

where *n* is the Hill exponent that is an empirical measure for cooperativity, and *K*_Hill_ is the effective dissociation constant.

### Determination of Hill exponents from the distribution of bound ComK

If *i* is the number of ligands to a single particle then $\left\langle i\right\rangle ={\sum }_{i=0}^{N}ip\left(i\right)$ is the average number of bound ligands to an ensemble of particles and $\left\langle {i}^{2}\right\rangle ={\sum }_{i=0}^{N}{i}^{2}p\left(i\right)$ is the second moment of the distribution $p\left(i\right)$ of bound ligands, and $N$ is the total number of possible binding sites. Following Wyman^41^ (combine Eqs. (8.2) and (9.1) therein), the Hill coefficient is given by

23 $$n=\frac{\left\langle {i}^{2}\right\rangle -{\left\langle i\right\rangle }^{2}}{\left\langle i\right\rangle \left(1-\left\langle i\right\rangle /N\right)}$$

The nominator of Eq. (23) is the variance of the observed distribution of bound ligands and the denominator is the variance of the corresponding binomial distribution. Since $p\left(i\right)$ is approximately accessible from the evaluation of particles in cryo-EM (Fig. 3b), the Hill coefficient was calculated form the cryo-EM statistics, using Eq. (23).

### The elastic-coupling model

We described the obtained coupling free energies (Fig. 4c) using a model derived by Rudnick and Bruinsma^18^. The model starts from the Hamiltonian for a two-dimensional worm-like chain (WLC)

24 $$H\left[\theta \left(s\right)\right]=\int {{\mathrm{d}}s}\left\{{k}_{\mathrm{B}}T\frac{{l}_{\mathrm{p}}}{2}{\left(\frac{{\mathrm{d}}\theta }{{\mathrm{d}}s}\right)}^{2}-f{\mathrm{cos}}\theta \right\}$$

Here, $s$ is the arclength of the chain, $\theta \left(s\right)$ is the angle between the chain and one coordinate, ${l}_{\mathrm{p}}$ is the persistence length, and $f$ is a tension applied to the rod. Solving Eq. (24) for small bending angles (${\cos }\theta \sim {\theta }^{2}/2$) and introducing two discontinuities (kinks) separated by Δ*s*, gives odd and even solutions that lead to an enthalpic contribution of the kinks

25 $$\Delta E=2{\alpha }^{2}\left({l}_{\mathrm{p}}/\xi \right)\left(1\pm {e}^{-\Delta s/\xi }\right){{\rm{in}}\; {\rm{units}}\; {\rm{of}}}\;{k}_{\mathrm{B}}T$$

Here, Δ*s* is the distance between the two kinks (protein binding sites), $\xi ={\left({k}_{\mathrm{B}}T{l}_{\mathrm{p}}/f\right)}^{1/2}$ is the decay length of tension along the DNA, ${l}_{\mathrm{p}}$ is the persistence length, and $\alpha$ is the local bending angle at the binding site. Compared to the enthalpic effect, the entropic contribution is rather small^18^ and was therefore omitted. Eq. (25) describes the effective change in binding energy of two proteins for the case, in which they introduce bending in DNA. Interestingly, Eq. (25) has a non-zero asymptotic limit at long distances between the binding sites, which accounts for the energy required to bend DNA by each protein separately. The plus sign is for the symmetric case, i.e., the two sites couple repulsively, which hampers binding of both proteins, and the minus sign indicates the attractive antisymmetric case. Clearly, the opposite is the case for ComK. Rather than introducing bending, binding of ComK reduces bending, which we interpret to be equivalent to a switch in signs. The helicity of DNA is incorporated via a cosine modulation between the signs in Eq. (25), thus leading to

26 $${\Delta g}_{J}=2{\alpha }^{2}\left({l}_{\mathrm{p}}/\xi \right)\left({1+{\cos }\left[\frac{2\pi }{\lambda }\left(\Delta s+\Delta {s}_{0}\right)\right]e}^{-\Delta s/\xi }\right)$$

for the coupling free energy. We used a similar expression to fit the change of Hill exponents with increasing spacer length

27 $$n=a\left({b+{\cos }\left[\frac{2\pi }{\lambda }\left(\Delta s+\Delta {s}_{0}\right)\right]e}^{-\Delta s/\xi }\right).$$

Here, $\lambda$ is the periodicity of DNA (10.5 bp), Δ*s* is the absolute spacer length, Δ*s*_0_ = 8 bp is the phase shift. The qualitative fractions of the EMSA were empirically fitted with a sum of a cosine and exponential function (solid line in Fig. 4d) with a periodicity of 10.5 bp, which resulted in a phase shift of Δ*s*_0_ = 7.2 ± 0.5 bp. When setting $\lambda$ = 10.5 bp and Δ*s*_0_ = 8 bp based on these results (Fig. 4d), a fit of the Hill exponents with Eq. (27) resulted in $\xi$ = 14 ± 8 bp (Fig. 4c left). To obtain the local bending angle, which can be interpreted as the change in bending in a box upon ComK binding, we used Eq. (26) and fixed the decay length to $\xi$ = 14, resulting in $\alpha$ = 24° ± 7° per box. The average change in bending across the full *comG* promoter (44 bp from box 1 to box 2) then gives a value of $2\alpha /$44 = 1.1° ± 0.3°/bp that can be compared to the result from cryo-EM (Fig. 3d, e).

### Gel retardation assay

The gel retardation assay, also termed EMSA, is capable of providing a qualitative estimate of ComK–DNA binding. The forward strand (Supplementary Table 7) and the reverse strand, at a final concentration of 10 μM were mixed in the following buffer: 50 mM Tris pH 7.5, 0.1 M NaCl, and 3 mM MgCl. The mixture was heated to 95 °C and slowly cooled in a PCR cycler, as previously described. The reaction mixture contained 25 nM dsDNA and 300 nM ComK, in the following buffer: 20 mM Tris pH 7.0, 50 mM l-Arg, 5 mM MgCl_2_, 150 mM KCl, 10 mM DTT, and 0.001% Tween-20. The reaction was incubated for 20 min and supplemented with glycerol to a final concentration of 10%. The reaction was loaded on a 5% native PAGE, composed of 4.2 ml of 30% polyacrylamide 29:1, 8.3 ml of 1.5 M Tris-HCl pH 8.8, 12.2 ml ddH_2_O, 150 μl of 10% ammonium persulfate, and 150 μl TEMED. The gels were ran in a Hoefer system at 100 V for 30 min, under TAE running buffer. The gels were than incubated in 100 ml ddH_2_O and a single drop of 0.625 mg/ml ethidium bromide, for 10 min. The gels were then washed and imaged with a Typhoon FLA 9500 scanner (GE). The fractions were computed by numerically integrating the bands in each lane, using Mathematica 11.2 (Wolfram).

### Sample preparation for cryo-EM

ComK at a concentration of 40 μM (500 μl) in 50 mM Tris pH 8.0, 6 M GdmCl, and 10 mM imidazole was injected on a HiTrap desalting column (5 ml, GE Healthcare) pre-equilibrated with 0.5 M arginine–HCl, pH 7.3. The protein containing fractions (32 μM) were then supplemented with 10 mM DTT to prevent the oxidation of the intrinsic cysteine residues of ComK. The ComK–DNA complexes were formed by quickly mixing 2.5 μl of ComK (32 μM) and 0.6 μl of DNA (20 μM) with 22 μl 20 mM Tris pH 7.0, 5 mM MgCl_2_, 150 mM KCl, 3 mM DTT, and 0.001% Tween-20. Immediately, after mixing, 2.5 μl of this mixture was transferred to UltrAuFoil R 1.2/1.3 300 mesh grids (Quantifoil). The grids were glow discharged before applying the sample, blotted for 2.5 s at 4 °C and 100% humidity, and plunge frozen in liquid ethane cooled by liquid nitrogen using a Vitrobot plunger (Thermo Fisher Scientific).

### Cryo-EM data acquisition

Cryo-EM data sets were collected on a Titan Krios G3i transmission electron microscope (Thermo Fisher Scientific) operated at 300 kV. ComK–DNA complexes featured preferred orientation, and therefore initial data sets collected at 0° tilt were used only to calculate binding statistics by 2D classification (Fig. 3a). To this end, 10,526, 3909, and 5108 0° tilt movies were recorded for the 8, 18, and 31 bp spacers, respectively. For 3D reconstructions, data were collected primarily at 40° tilt. The 8 bp spacer data set comprised 6537 40° tilt movies. The 18 bp spacer data set comprised 8024 40° tilt movies and 431 0° tilt movies. Movies were recorded in counting mode on a K3 direct detector (Gatan) at the end of BioQuantum energy filter (Gatan), using a slit of 20 or 15 eV at a nominal magnification of 105,000×, which corresponds to a physical pixel size of 0.86 Å. The dose rate was set to 20.6 e^−^/pixel/s and the total exposure time was 2 s, resulting in an accumulated dose of 55.7 e^−^/Å^2^. Each movie was fractionated into 50 frames of 0.04 s. The nominal defocus range was −0.8 to −1.3 μm; however, the actual defocus range was larger because of tilting. Imaging was done using an automated low-dose procedure implemented in SerialEM^68^, in which a single image was collected from the centre of each hole. Image shift was used to target an array of up to six neighbouring holes along the tilt axis (depending on hole array orientation), and stage shift to move between arrays. Beam tilt was adjusted to achieve coma-free alignment when applying image shift.

### Single-particle cryo-EM image processing

Image processing was performed using CryoSPARC software^69^. Movie frames were aligned using patch motion correction, followed patch CTF estimation. A total of 4024 micrographs of the 8 bp spacer data set, which featured a resolution better than 10.0 Å, relative ice thickness lower than 1.4, and full frame motion correction smaller than 100 pixels were selected for further processing. The processing procedure is outlined in Supplementary Fig. 5. An initial particle data set was created by manual picking, followed by 2D classification and automated picking, using the newly generated 2D classes as templates. About 130,000 automatically picked particles were extracted, binned 4 × 4 (110-pixel box size, 3.44 Å/pixel), and subjected to multiple rounds of 2D classification in order to clean the data set. Particles were separated into ComK–DNA complexes and free DNA (100,695 and 9500 particles, respectively), followed by ab initio 3D reconstruction of the ComK–DNA particles with two 3D classes. The higher-resolution 3D class (62,177 particles) was refined using the unbinned data (440 box size) to a final resolution of 6.6 Å.

For the 18 bp spacer, 7450 movies were retained using the same criteria as above (Supplementary Fig. 6). Automated picking resulted in ~1,361,000 particles, which were cleaned by 2D classification and separated into two data sets, ComK–DNA and free DNA, comprising 422,425 and 29,171 particles, respectively. Ab initio 3D reconstruction followed using 4 × 4 binned images (3.44 Å/pixel). Both the ComK–DNA and free DNA particles were classified in 3D based on particle curvature, resulting in three well-resolved classes in each data set. In addition, in an attempt the find a homogeneous subset that will yield the highest possible resolution, the ComK–DNA particles were classified in 3D sequentially, as depicted in Supplementary Fig. 6. All 3D classes were refined by homogenous refinement using the unbinned data and 440-pixel boxes. Final resolutions and angular distributions can be seen in Supplementary Fig. 7. 3D visualization was performed using UCSF Chimera^70^ (Version 1.12) or ChimeraX^71^ (Version 0.92). The resulting 3D maps show a plane that connects the surface of a ComK dimer in the first box with that of the second box. In addition, the peripheral surface of the ComK dimers that faces away from the DNA appear distorted in the maps. This indicates that the ComK–DNA complexes are adsorbed to the water–air interface via this surface (Supplementary Fig. 10).

Binding stoichiometry of ComK to DNA was determined using the initial 0° tilt data sets, as described above. Movie frames were aligned using patch motion correction, followed CTF estimation, and the highest quality micrographs were retained. A total of 10,526, 3909, and 4982 micrographs were retained in the 8, 18, and 31 bp spacer data sets, respectively. Initial 2D classed were generated by manual picking, followed by automated picking using the newly generated 2D templates. The number of complexes in each group was determined by 2D classification after discarding classes that did not resolve well. In total, the 8, 18, and 31 bp spacer data sets comprised 508,389, 22,6121, and 231,710 particles, respectively.

### Calculation of curvature angles of free and ComK-bound comG

Curvature angles (Fig. 3d, e) were computed from a coarse-grained spline that was determined from the EM maps of free DNA and of the ComK–*comG* complex. The EM maps were first binned (four *z*-slices per bin for free DNA, three *z*-slices per bin for ComK–*comG*) and subsequently fit with a Bezier 3D-spline, using Mathematica 11.2 (Wolfram). The spline was subsequently divided into segments of *b* = 0.34 nm length. The resulting coordinates ${{\bf{u}}}_{i}$ were used to compute the vector ${{\bf{v}}}_{i}$ of each segment *i* via ${{\bf{v}}}_{i}={{\bf{u}}}_{i+1}-{{\bf{u}}}_{i}$. The angle between neighboured segments was finally computed according to ${\theta }_{i}={{{\cos }}}^{-1}{{\bf{v}}}_{i+1}{{\bf{v}}}_{i}/{b}^{2}$.

### DNA model building and refinement

The structures of the two promotors *addAB* and *comG* in complex with ComK were built and refined, using the respective cryo-EM maps. Idealized B-DNA fibre-like double helix models were produced using Coot^72^ with the sequence of the promoters *addAB* (78 bp in total) with a spacer of 8 bp between boxes, and *comG* (93 bp in total) with a spacer of 18 bp. The resulting straight DNA duplexes were used as starting models for alignment to their respective density maps, using manual fitting and real-space rigid-body refinement with the whole duplex defined as one group, leading to two idealized models of opposite direction for both promoters. The directional uncertainty of the models along the long axis of the EM map was solved by accounting for the asymmetry of the sequences at the 5′- and 3′-ends relatively to the binding boxes. However, whereas the size of the EM envelope of the *addAB* promoter at reasonable sigma value (typically 3.5–5.0) corresponded well to the length and diameter of the corresponding idealized model (~260 and 20 Å respectivelly), the EM map of the longer promoter, *comG*, was too short by ~30 Å (10 bp) to accommodate a 93 bp double helix with an expected length of ~310 Å. Most probably the longer DNA-end of the promoter was more flexible than the rest of the duplex. As a result, 10 bp are missing in the model of the *comG* structure. The aligned models were then submitted to a second real-space rigid-body cycle. To this end, the number and size of the rigid-body groups were determined by identifying the base pairs for which the DNA duplex deviated from the straight initial model. These parts were then adapted to the EM map, using Phenix^73^. This step led to correlation coefficients (CC) of CC(mask) = 0.35 and CC(box) = 0.48 (from 0.11 and 0.30, respectively) for the whole *addAB* duplex. Subsequent cycles of manual corrections with Coot^72^ and real-space refinement with Phenix^73^ led to the final values of CC(mask) = 0.74 and CC(box) = 0.66. For the refinement of the *comG* model, successive cycles of manual corrections and real-space rigid-body refinement were entirely conducted with Coot^72^. The final real-space refinements of this incomplete model (84 bp out of 93 bp) using Phenix^73^ led to the final values of CC(mask) = 0.75 and CC(box) = 0.63. For both structures, the refinement cycles with Phenix^73^ included restraints on bond lengths and bond angles, on Watson–Crick base-pairing H-bonds, planarity, parallelism, and on stacking. Despite the low resolution, the atomic displacement parameters (ADPs or *B*-factors) were refined during the last cycle of refinement. Remarkably, in both structures, the lowest values were obtained for the nucleotides forming A-tracts targets of the ComK monomers. During the entire process, the progress was monitored by systematic inspection of the models in their respective EM map using Coot^72^ and validated using MolProbity^74^. Assuming that two ComKs bind their respective box in a similar manner, we were able to position the asymmetrically bent DNA double helices along the long axis of the map with a level of uncertainty of ±2 bp. The refinement statistics are given in the Supplementary Table 5.

### Comparison of the DNA model with smFRET

Predictions of mean FRET values for the ComK–*addAB* complex were done by first simulating possible dye positions (${{\bf{s}}}_{i}$, ${{\bf{t}}}_{j}$) along with their probabilities (${p}_{i}$,${q}_{j}$) for donor ($i$) and acceptor ($j$) fluorophores. The FRET efficiency was then evaluated as $E={\sum }_{i}{\sum }_{j}\frac{{R}_{0}^{6}}{{R}_{0}^{6}+{r}_{{ij}}^{6}}{p}_{i}{q}_{j}$ where ${r}_{{ij}}=|{{\bf{s}}}_{i}-{{\bf{t}}}_{j}|$ and ${R}_{0}=5.4\,{\mathrm{nm}}$ is the Förster distance. The values for ${{\bf{s}}}_{i}$, ${{\bf{t}}}_{j}$, ${p}_{i}$, and ${q}_{j}$ were obtained by modelling the fluorophores as bulky spheres, which were attached to an adenine base in the following manner: a nitrogen atom was positioned at a distance of 132 pm from the C8-atom of the adenine base of the atomic model of the *addAB* promoter (see previous paragraph). The atom was placed in the adenine plane at a N–C8–N9 angle of 123°. The fluorophore was a attached to this N-atom via a polyethylene chain of seven C-atoms. The conformational freedom of the linker was accounted for using the rotational isomeric state model^75^: each bond along the chain samples the anti, gauche+ and gauche− rotamers. Chains that sterically clashed^76^ with the complex were discarded. However, the linker was seriously sterically constraint by the tight packing of bases and the DNA backbone, which is unlikely to be precise in its details given the low resolution of our 3D-EM maps that does not allow us to correctly model tilt and buckling of the base pairs. In addition, it cannot be excluded that the dye attachment causes local distortions of the DNA that are not reflected in the atomic model. Given the resulting steric constraints, we performed a set of simulations in which we allow clashes of linker atoms with the DNA. Three sets were considered, clashes of only the first linker atom, clashes of the first four linker atoms, and clashes of the first seven linker atoms (Supplementary Fig. 8 and Supplementary Table 6). Geometric and energetic parameters of polyethylene were taken from Rehan et al.^75^, and the fluorophore size was taken from Kalinin et al.^77^. The excluded volume of ComK was taken into account. To this end, the EM map of ComK given on a 3D grid with 1.718 Å spacing was used. We performed calculations for two different contour levels of the EM map for ComK, leading to volumes of 19.6 and 47.8 nm^3^ for a ComK dimer. The volumes were filled with atoms of van der Waals radius of 0.859 Å. The results are summarized in Supplementary Table 6. For comparison, with an average amino acid volume^78^ of 0.13 nm^3^ and a length of a ComK monomer of 195 amino acids, we expect a volume of 50.7 nm^3^ for a ComK dimer.

### Reporting summary

Further information on research design is available in the Nature Research Reporting Summary linked to this article.

## Supplementary information

Supplementary Information

Reporting Summary

## Data Availability

Data supporting the findings of this manuscript are available from the corresponding authors upon reasonable request. A reporting summary for this article is available as a Supplementary Information file. Cryo-EM maps and atomic coordinates have been deposited in the Electron Microscopy Data Bank (EMDB) and Protein Data Bank (PDB). The accession codes are EMD-11022 and 6Z0S for the *comG*–ComK complex (18 bp spacer) and EMD-12260 and 7NBN for the *addAB*–ComK complex (8 bp spacer). The single-molecule raw data were deposited on the Zenodo data repository under the 10.5281/zenodo.4468372. Source data are provided with this paper.

## Source data

Source Data

## Code availability

The single-molecule analysis code is accessible as a custom WSTP add-on for Mathematica (Wolfram Research) at https://schuler.bioc.uzh.ch/programs/.
