# Supplementary material for: Allostery through DNA drives phenotype switching
Source: Nat Commun. 2021 May 20;12:2967. doi: 10.1038/s41467-021-23148-2 (PMC8170675; doi:10.1038/s41467-021-23148-2)
Supplement: Supplementary file 1 — Supplementary Information [file 41467_2021_23148_MOESM1_ESM.pdf]

Supplementary Figures

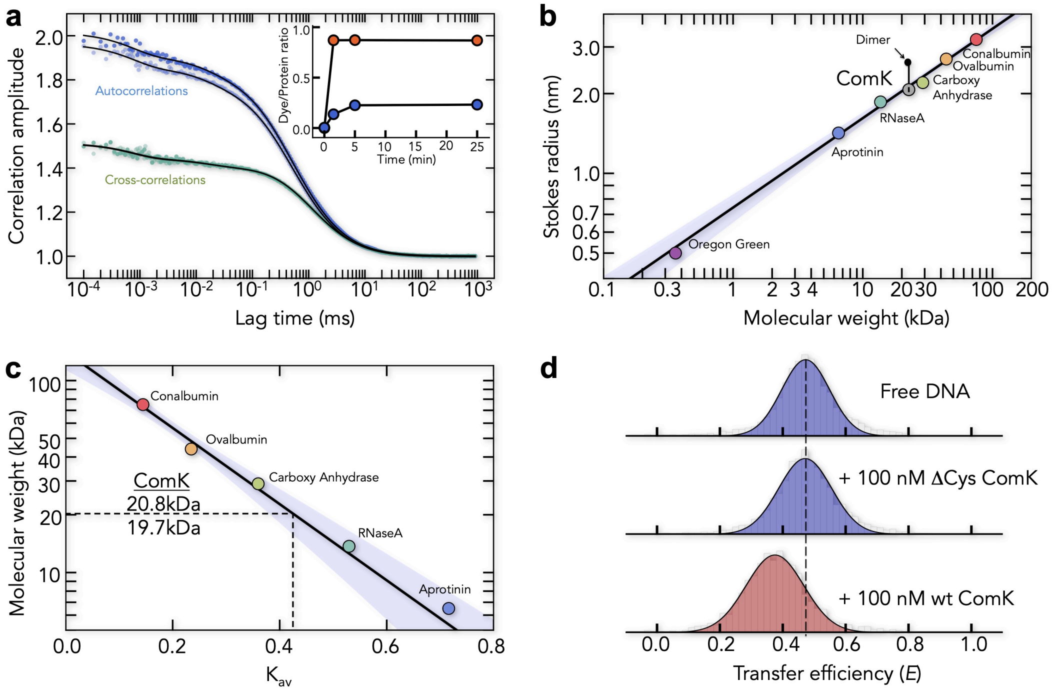

Supplementary Fig. 1. ComK is a monomer in solution. (**a**) 2fFCS auto- and cross-correlation functions of singly labelled ComK and fits with a model containing two triplet relaxation processes and one decay for the diffusion of ComK. Inset: Labelling kinetics of wildtype ComK (wt ComK, blue circles) and a variant of ComK with an additional cysteine at the N-terminus (N-Cys ComK, red circles, see Supplementary Table 3) with 0.9 mol equivalents of AlexaFluor 488, demonstrating predominantly specific labelling of the N-terminal cysteine. (**b**) Stokes radius of ComK (grey) in comparison with the Stokes radii of our calibration standards. Based on the measured Stokes radius of ComK, we estimated the radius of a ComK dimer assuming a spherical shape (black dot). Solid line is a fit with a power law resulting in an exponent of 0.34 ± 0.02 and an amplitude of 0.74 ± 0.04 where the variation is the error of the fit. The Stokes radius of ComK is 2.07 ± 0.03 nm based on three technical replicates. (**c**) Molecular mass determination using size-exclusion chromatography. The molecular weight of five standard proteins is plotted as function of the relative elution volume. Solid line is a fit with $MW = 10^{(m\,Kav + n)}$ with m = 2.0 ± 0.2 and n = 2.15 ± 0.04. The variation is the error of the fit. The calculated molecular masses of ComK, obtained in two experiments at 3 and 4 μM, are indicated. (**d**) A variant of ComK with native cysteines replaced by alanine (ΔCys ComK) is incapable of binding to DNA. The *comG* promoter sequence was labelled at box 2 (Supplementary Table 2). 30 pM DNA (top) was mixed with 100 nM of ΔCys ComK (middle) and wildtype (wt) ComK (bottom). The dashed line is centred at the FRET histogram of the free DNA.

4

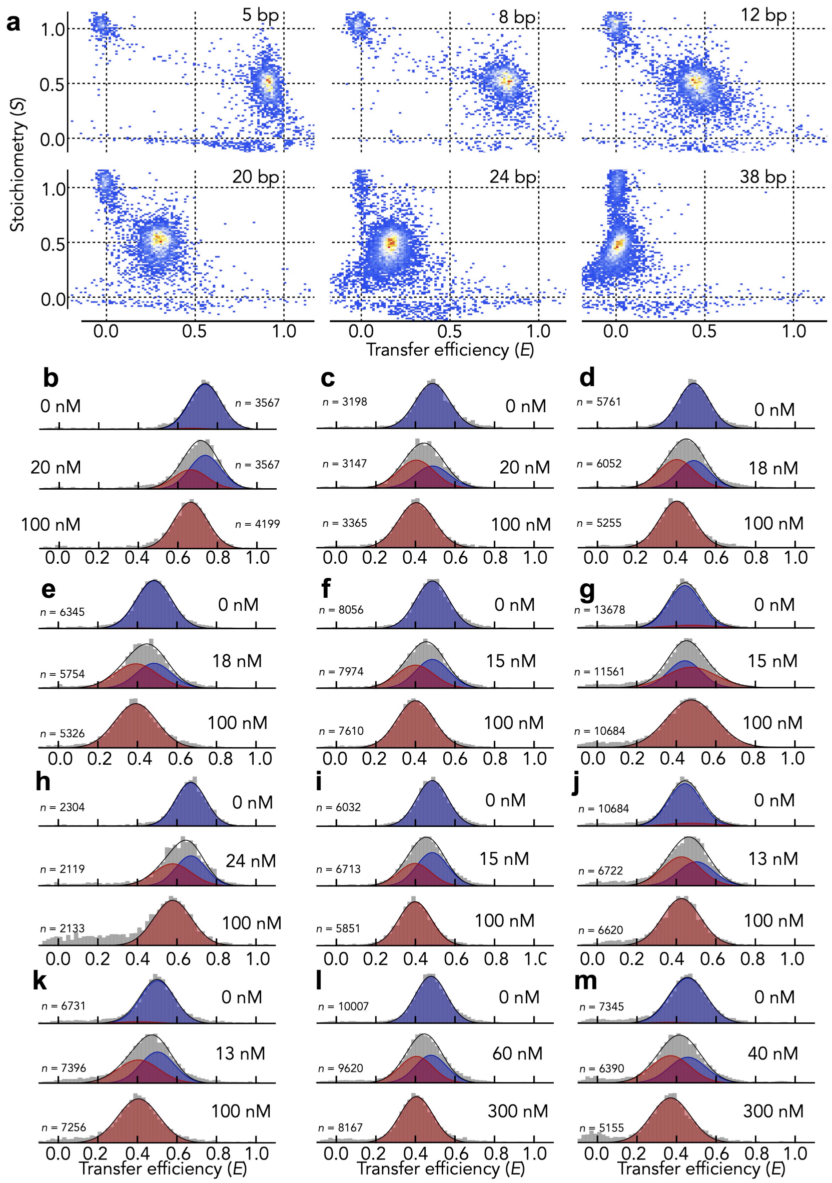

Supplementary Fig. 2. Representative FRET histograms. (**a**) FRET- and Stoichiometry-histograms for selected *comG*-labeling constructs (Supplementary Table 1) at pH7. The base-pair separation between donor and acceptor is indicated. FRET-histograms are also shown for the promoters *addAB* with labelled box 1 (**b**), *addAB* with labelled box 2 (**c**), *comG* with 55% GC content in the spacer (**d**), *comG* with 72% GC content in the spacer (**e**), *comG* with mismatch in the spacer (**f**), *comG* with a nick in the spacer (**g**), *comK* promoter (**h**), artificial promoter with 8 bp spacers labelled in box 2 and containing 2 boxes (**i**), 3 boxes (**j**), and 4 boxes (**k**), the isolated box 1 of *comG* (**l**), and the isolated box 2 of *comG* (**m**). Unless otherwise state, box 1 is labelled.

5

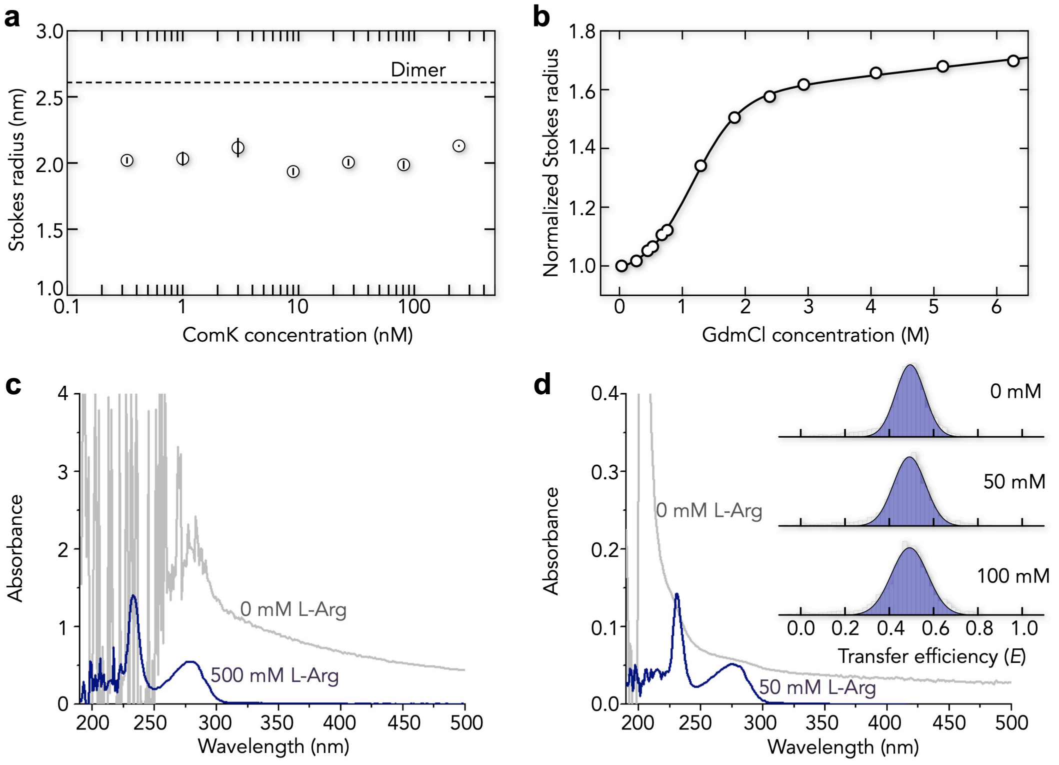

Supplementary Fig. 3. The dimerization affinity and stability of ComK is low. (**a**) The Stokes radius of ComK at increasing concentrations of unlabelled ComK does not increase, indicating that ComK is a monomer under our experimental conditions. Error bars represent the variation of three technical replicates. (**b**) The Stokes radius relative to the radius in the absence of GdmCl is shown as function of the denaturant (GdmCl) concentration. Solid line is a fit with a two-state folding equilibrium that results in a free energy difference between folded and unfolded ComK of -8.8 ± 0.3 kJ/mol (variation is the error of the fit), suggesting that 2.4 ± 0.7 % of the molecules are unfolded under physiological conditions, which explains the strong aggregation tendency of ComK. (**c**, **d**) Absorbance spectra show that ComK forms aggregates at 22 μM (**c**) and 2 μM (**d**) ComK-concentration (grey lines). However, the wavelength-dependent light-scattering decay is absent in the presence of L-Arg in concentrations of 500 mM (**c**) and 50 mM (**d**). Inset: FRET histograms of labelled *comG* promoter at different L-Arg concentrations (indicated).

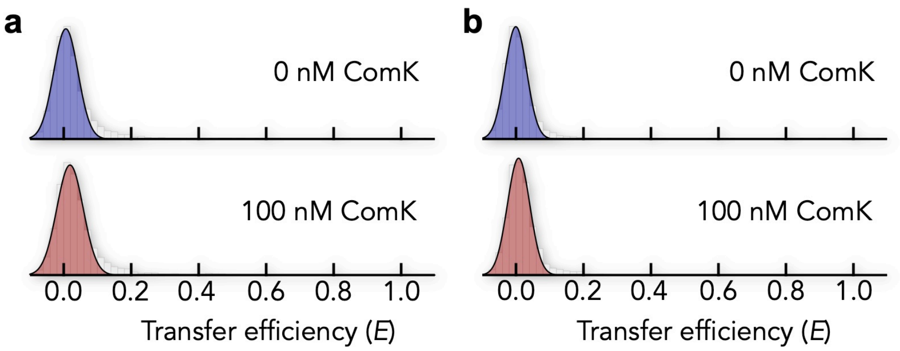

Supplementary Fig. 4. FRET histograms of the terminally labelled *comG*-promoter. The acceptor was placed 5’ of box 1 whereas the donor was placed 3’ of box2. The donor-acceptor sequence separation is 45 bp (a) and 69 bp (b).

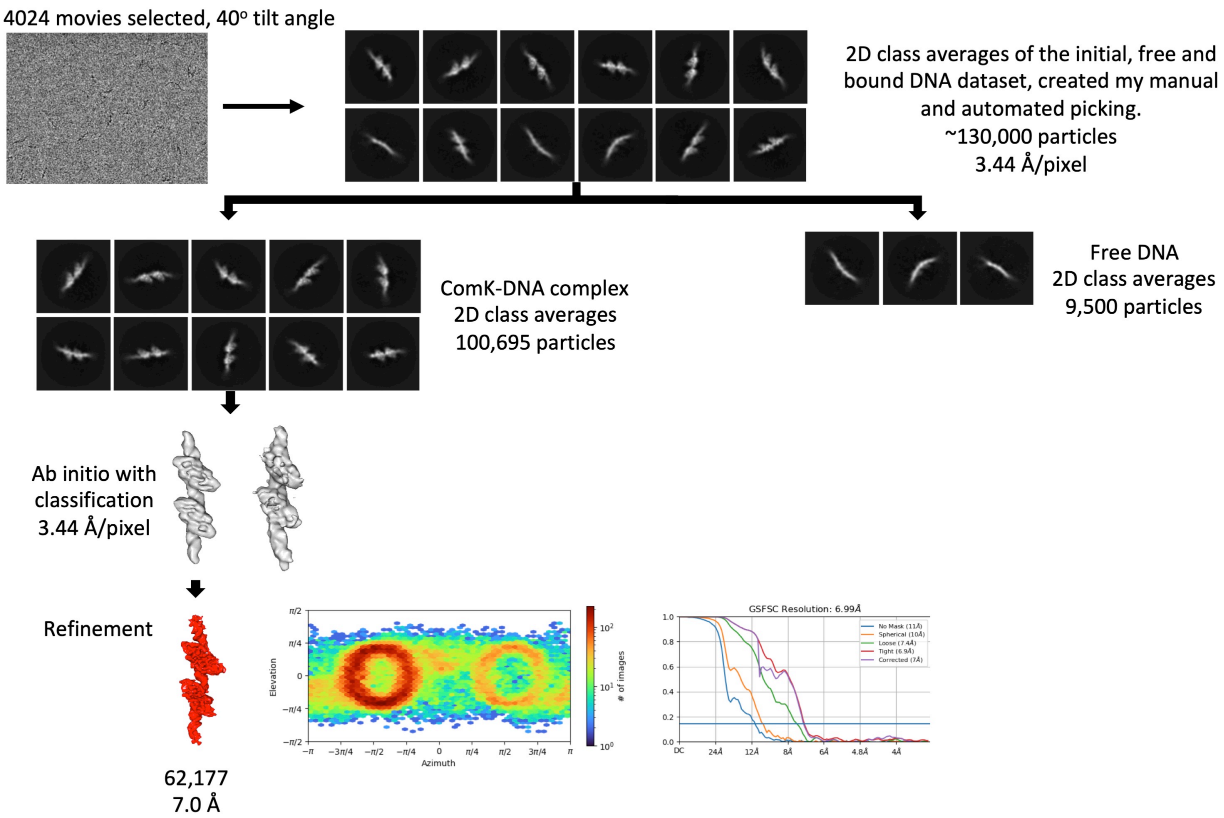

Supplementary Fig. 5. Scheme for single particle reconstruction of the 8 bp data set. The details of the process are described in the Methods section. Briefly, a mixed particle data set was first extracted from the micrographs, containing both free and ComK-bound DNA complexes. This mixed data set was cleaned and separated into two data sets, ComK-DNA and free DNA, by iterative 2D classification. Processing, followed by ab initio 3D reconstruction and classification, and refinement of the best 3D class. Angular distribution and FSC curves are presented for the final 3D map.

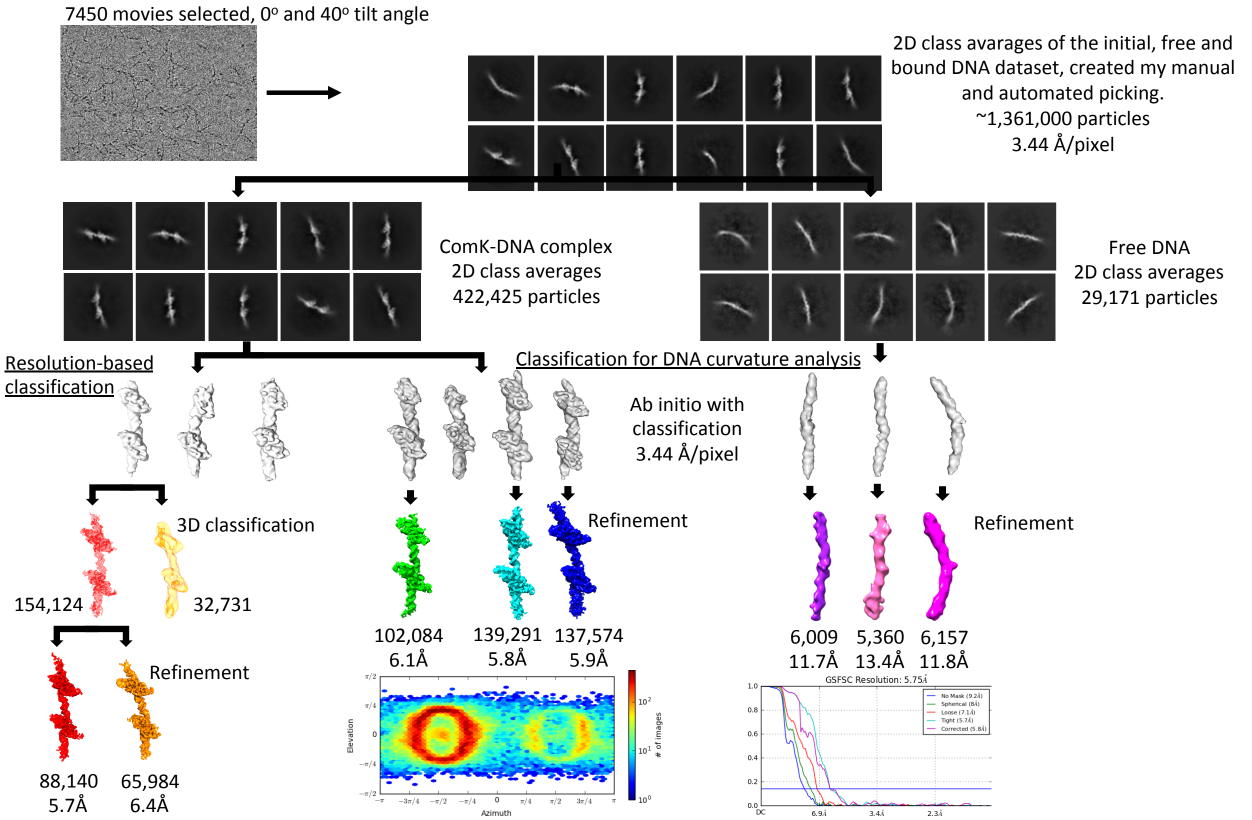

Supplementary Fig. 6. Scheme for single particle reconstruction of the 18 bp data set. The details of the process are described in the Methods section. Briefly, a mixed particle data set was first extracted from the micrographs, containing both free and ComK-bound DNA complexes. This mixed data set was cleaned and separated into two data sets, ComK-DNA and free DNA by iterative 2D classification. Processing followed by ab initio 3D reconstruction and classification. The ComK-DNA and free DNA were both classified in 3D based on DNA curvature. In addition, the ComK-DNA model was iteratively classified to achieve the best resolution. The map coloured in red (5.7 Å resolution) was used for atomic modelling of the DNA molecule and appears in Fig. 3c. The six refined maps, which were classified based on DNA curvature, were used for the curvature analysis in Fig. 3d and e. Angular distribution and FSC curves are presented for the final 3D map.

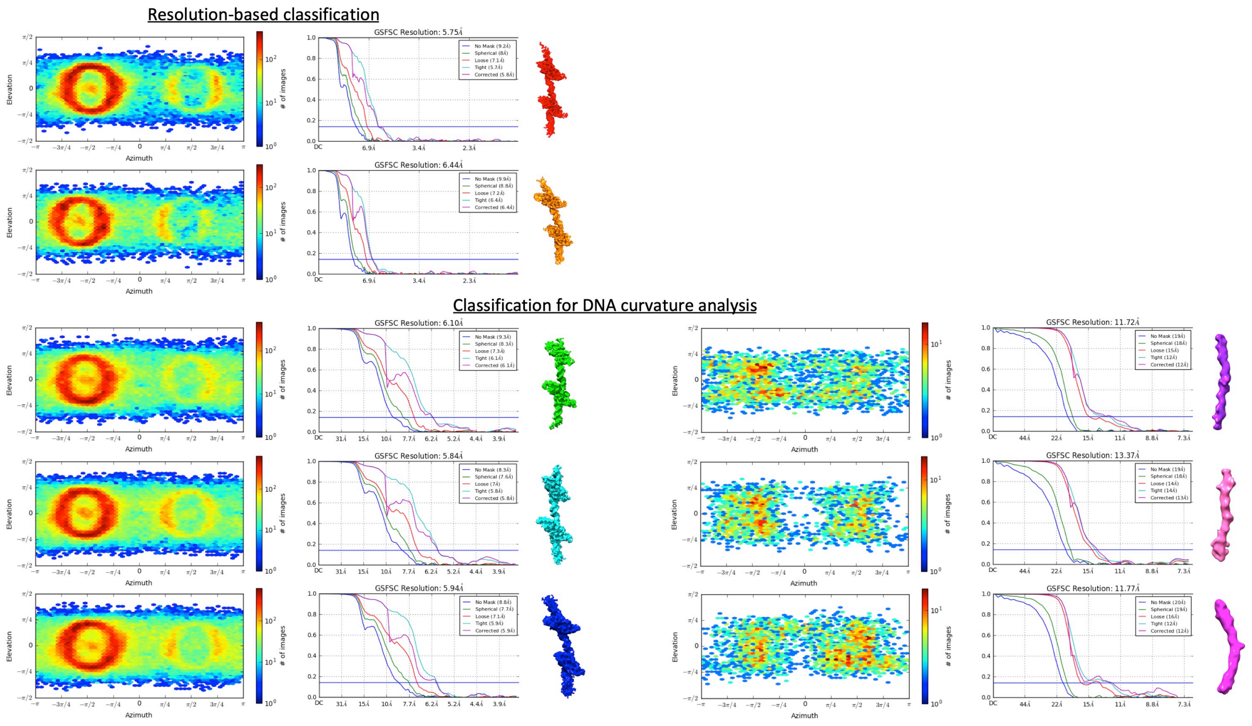

Supplementary Fig. 7. Angular distribution and FSC curves of the refined maps from the 18 bp spacer data set. Maps are coloured the same as in Supplementary Fig. 6 above.

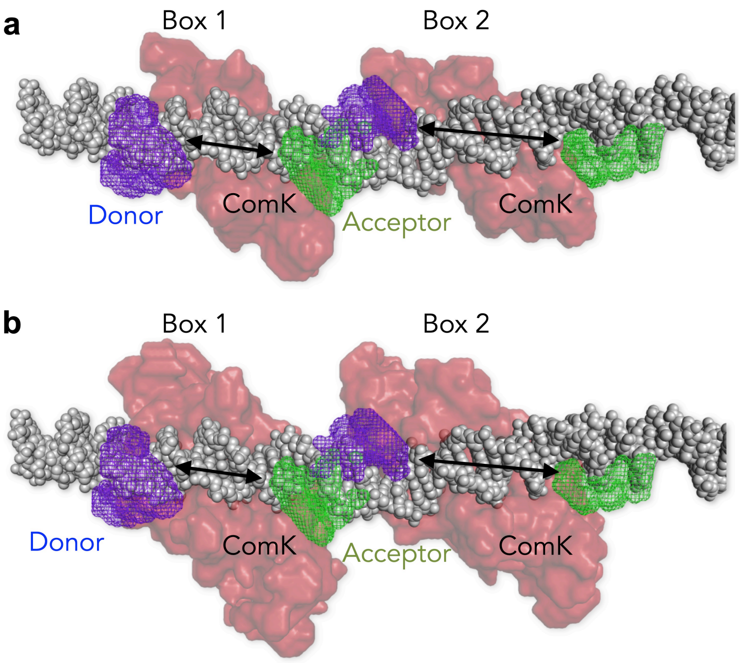

Supplementary Fig. 8. Simulation of the dye spatial distribution on the *addAB* promoter. (**a**) Atomistic model of the *addAB* promoter including the EM-density of the ComK dimers (red, 19.6 nm^3 each dimer). The positional distributions for donor and acceptor in the boxes are shown in purple and green, respectively. Clashes of the first four linker-atoms were ignored in the simulation (see Supplementary Table 6). (**b**) Same as **a** but for a larger volume of a ComK dimer (47.8 nm^3).

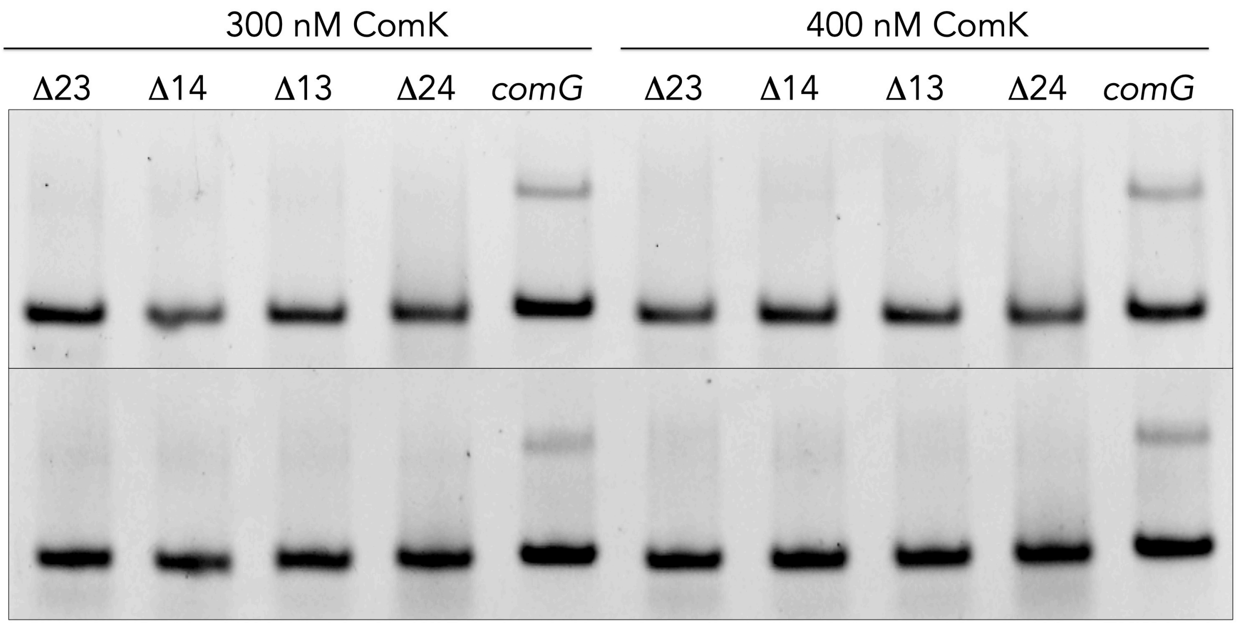

Supplementary Fig. 9. Gel retardation experiment (EMSA) of DNA-constructs with a single A-tracts in a binding box. The constructs are based on the *comG* promoter (Supplementary Table 5). Upper and lower panel are two independent repetitions of the same experiment. The concentration of ComK is indicated.

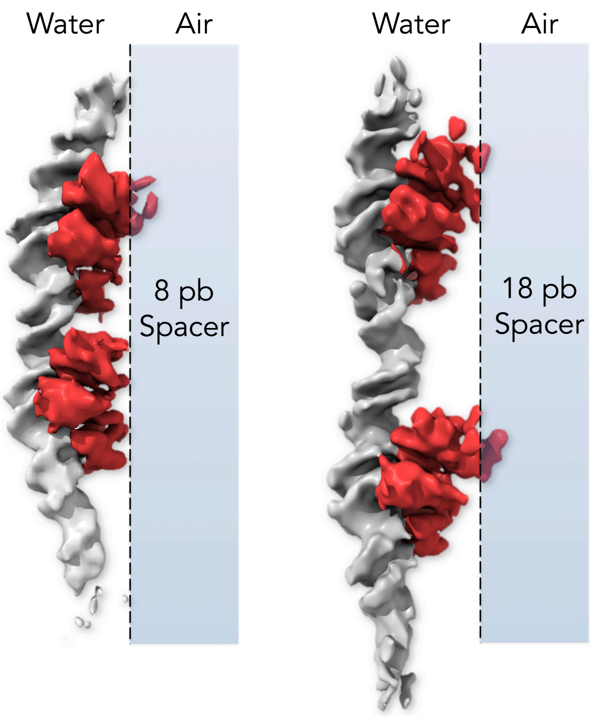

Supplementary Fig. 10. Potential air-water interface of the ComK-DNA complex with 8 bp and 18 bp spacer. The 3D-maps indicate a plane that connects the peripheral surfaces of ComK dimers (red) in the boxes.

Supplementary Table 1. List of labelled DNA for smFRET experiments that monitor the curvature in the *comG* promoter. The two AT boxes are shaded in cyan. Donor and acceptor attachment sites are shown in green and red, respectively. Duplexes were obtained from combinations of the three Watson strands (W) with the four Crick strands (C), thus resulting in the constructs shown in Fig. 2b.

comG	Length	Sequence
**W71**	84	CAGTTGAAAGTCTTTTTTCTTGCCAGAAAGAATTGGTTTTTCAGCATATAACATCTCACAAAATCACGTT**T**TCCCTGTTTGATT
W64	84	CAGTTGAAAGTCTTTTTTCTTGCCAGAAAGAATTGGTTTTTCAGCATATAACATCTCACAAAA**T**CACGTTTTCCCTGTTTGATT
W41	84	CAGTTGAAAGTCTTTTTTCTTGCCAGAAAGAATTGGTTTT**T**CAGCATATAACATCTCACAAAATCACGTTTTCCCTGTTTGATT
C66	84	AATCAAACAGGGAAAACG**T**GATTTTGTGAGATGTTATATGCTGAAAAACCAATTCTTTCTGGCAAGAAAAAAGACTTTCAACTG
C62	84	AATCAAACAGGGAAAACGTGAT**T**TTGTGAGATGTTATATGCTGAAAAACCAATTCTTTCTGGCAAGAAAAAAGACTTTCAACTG
C58	84	AATCAAACAGGGAAAACGTGATTTTG**T**GAGATGTTATATGCTGAAAAACCAATTCTTTCTGGCAAGAAAAAAGACTTTCAACTG
C43	84	AATCAAACAGGGAAAACGTGATTTTGTGAGATGTTATATGC**T**GAAAAACCAATTCTTTCTGGCAAGAAAAAAGACTTTCAACTG
C32	84	AATCAAACAGGGAAAACGTGATTTTGTGAGATGTTATATGCTGAAAAACCAA**T**TCTTTCTGGCAAGAAAAAAGACTTTCAACTG

Supplementary Table 2. List of labelled DNA for smFRET ComK-binding experiments. The two AT boxes are highlighted in cyan. Donor and acceptor attachment sites are shown in green and red, respectively. For the constructs of the isolated boxes, we added a flanking region (15-17 bp) before and after each box with GC pairs at the termini to prevent helix fraying. The construct denoted by *Design 18bp* is identical to *comG: box 1 labelled*. It had been measured twice in duplicates (see Supplementary Table 4).

Promoter	Length	Sequence
***comG*: box 1 labelled**	93	CAGTTGAAAGTCTTTTTTCTTGCCAGAAAGAATTGGTTTTTCAGCATATAACATCTCACAAAATCACGTTTTCCCTGTTTGATTACCTTTTCT<br>GTCAACTTTCAGAAAAAAGAACGGTCTTTCTTAACCAAAAAGTCGTATATTGTAGAGTGTTTTAGTGCAAAAGGGACAAACTAATGGAAAAGA
***comG*: spacer labelled**		CAGTTGAAAGTCTTTTTTCTTGCCAGAAAGAATTGGTTTTTCAGCATATAACATCTCACAAAATCACGTTTTCCCTGTTTGATTACCTTTTCT<br>GTCAACTTTCAGAAAAAAGAACGGTCTTTCTTAACCAAAAAGTCGTATATTGTAGAGTGTTTTAGTGCAAAAGGGACAAACTAATGGAAAAGA
***comG*: box 2 labelled**	93	CAGTTGAAAGTCTTTTTTCTTGCCAGAAAGAATTGGTTTTTCAGCATATAACATCTCACAAAATCACGTTTTCCCTGTTTGATTACCTTTTCT<br>GTCAACTTTCAGAAAAAAGAACGGTCTTTCTTAACCAAAAAGTCGTATATTGTAGAGTGTTTTAGTGCAAAAGGGACAAACTAATGGAAAAGA
***comG*: isolated box 1 labelled**	46	CTTTTTTCTTGCCAGAAAGAATTGGTTTTTCAGCATATAACATCTC<br>GAAAAAAGAACGGTCTTTCTTAACCAAAAAGTCGTATATTGTAGAG
***comG*: isolated box 2 labelled**	43	CATATAACATCTCACAAAATCACGTTTTCCCTGTTTGATTACC<br>GTATATTGTAGAGTGTTTTAGTGCAAAAGGGACAAACTAATGG
***comG* GC-55%: box 1 labelled**	93	CAGTTGAAAGTCTTTTTTCTTGCCAGAAAGAATTGGTTTTTCAGCAGCTAACAGCTCACAAAATCACGTTTTCCCTGTTTGATTACCTTTTCT<br>GTCAACTTTCAGAAAAAAGAACGGTCTTTCTTAACCAAAAAGTCGTCGATTGTCGAGTGTTTTAGTGCAAAAGGGACAAACTAATGGAAAAGA
***comG* GC-72%: box 1 labelled**	93	CAGTTGAAAGTCTTTTTTCTTGCCAGAAAGAATTGGTTTTTCGGCCGCTAACGGCTCACAAAATCACGTTTTCCCTGTTTGATTACCTTTTCT<br>GTCAACTTTCAGAAAAAAGAACGGTCTTTCTTAACCAAAAAGCCGGCGATTGCCGAGTGTTTTAGTGCAAAAGGGACAAACTAATGGAAAAGA
***comG* Mismatch: box 1 labelled**	93	CAGTTGAAAGTCTTTTTTCTTGCCAGAAAGAATTGGTTTTTCAGCAATATACATCTCACAAAATCACGTTTTCCCTGTTTGATTACCTTTTCT<br>GTCAACTTTCAGAAAAAAGAACGGTCTTTCTTAACCAAAAAGTCGTATATTGTAGAGTGTTTTAGTGCAAAAGGGACAAACTAATGGAAAAGA
***comG* Nicked: box 1 labelled**	50<br>43<br>93	CAGTTGAAAGTCTTTTTTCTTGCCAGAAAGAATTGGTTTTCAGCATATA<br>ACATCTCACAAAATCACGTTTTCCCTGTTTGATTACCTTTTCT<br>GTCAACTTTCAGAAAAAAGAACGGTCTTTCTTAACCAACAAGTCGTATATTGTAGAGTGTTTTAGTGCACAAGGGACAAACTAATGGAAAAGA
***comK*: box 1 labelled**	78	CTTATGAAAGTAAAATCGGTTTATTACTAGTCATTTAGTACCATTAAATATCATTAAAAGATGATTTTATCTTAAATG<br>GAATACTTTCATTTTAGCCAAATAATGATCAGTAAATCATGGTAATTTATAGTAATTTTCTACTAAAATAGAATTTAC
***addAB1*: box 1 labelled**	57	GATGTGCGGAGATAATCAGCTTTTTATATGTGAAAAGGCCGTTTTTACCAATAGATC<br>CTACACGCCTCTATTAGTCGAAAAATATACACTTTTCCGGCAAAAATGGTTATCTAG
***addAB2*: box 2 labelled**	57	GATGTGCGGAGATAATCAGCTTTTTATATGTGAAAAGGCCGTTTTTACCAATAGATC<br>CTACACGCCTCTATTAGTCGAAAAATATACACTTTTCCGGCAAAAATGGTTATCTAG
addAB 2 boxes	57	GATGTGCGGAGAAAAGGCCGTTTTTATATGTGAAAAGGCCGTTTTTACCAATAGATC<br>CTACACGCCTCTTTTCCGGCAAAAATATACACTTTTCCGGCAAAAATGGTTATCTAG
addAB 3 boxes	78	GATGTGCGGAGAAAAGGCCGTTTTTATATGTGAAAAGGCCGTTTTTATATGTGAAAAGGCCGTTTTTACCAATAGATC<br>CTACACGCCTCTTTTCCGGCAAAAATATACACTTTTCCGGCAAAAATATACACTTTTCCGGCAAAAATGGTTATCTAG
addAB 4 boxes	99	GATGTGCGGAGAAAAGGCCGTTTTTATATGTGAAAAGGCCGTTTTTATATGTGAAAAGGCCGTTTTTATATGTGAAAAGGCCGTTTTTACCAATAGATC<br>CTACACGCCTCTTTTCCGGCAAAAATATACACTTTTCCGGCAAAAATATACACTTTTCCGGCAAAAATATACACTTTTCCGGCAAAAATGGTTATCTAG
Designed 8bp	78	GAAAGTCTTTTTTCTTGCCAGAAAGAATTGGTTTTT TATATGTGAAAATCACGTTTTCCCTGTTTGATTACCTTTTCT<br>CTTTCAGAAAAAAGAACGGTCTTTCTTAACCAAAAA ATATACACTTTTAGTGCAAAAGGGACAAACTAATGGAAAAGA
Designed 14bp	84	GAAAGTCTTTTTTCTTGCCAGAAAGAATTGGTTTTTCAGCATATAACATCAAAATCACGTTTTCCCTGTTTGATTACCTTTTCT<br>CTTTCAGAAAAAAGAACGGTCTTTCTTAACCAAAAAGTCGTATATTGTAGTTTTAGTGCAAAAGGGACAAACTAATGGAAAAGA
Designed 18bp	93	GAAAGTCTTTTTTCTTGCCAGAAAGAATTGGTTTTTCAGCATATAACATCTCACAAAATCACGTTTTCCCTGTTTGATTACCTTTTCT<br>CTTTCAGAAAAAAGAACGGTCTTTCTTAACCAAAAAGTCGTATATTGTAGAGTGTTTTAGTGCAAAAGGGACAAACTAATGGAAAAGA
Designed 24bp	94	GAAAGTCTTTTTTCTTGCCAGAAAGAATTGGTTTTTCAGCATATAACATCTCACCAGCATAAAATCACGTTTTCCCTGTTTGATTACCTTTTCT<br>CTTTCAGAAAAAAGAACGGTCTTTCTTAACCAAAAAGTCGTATATTGTAGAGTGGTCGTATTTTAGTGCAAAAGGGACAAACTAATGGAAAAGA
Designed 31bp	101	GAAAGTCTTTTTTCTTGCCAGAAAGAATTGGTTTTTTACTAGTCATTTAGTACCATTAAATATCATTAAAATCACGTTTTCCCTGTTTGATTACCTTTTCT<br>CTTTCAGAAAAAAGAACGGTCTTTCTTAACCAAAAAATGATCAGTAAATCATGGTAATTTATAGTAATTTTAGTGCAAAAGGGACAAACTAATGGAAAAGA

Supplementary Table 3. Amino acid sequences of the ComK constructs used in this study. The codon optimized construct contained a His_6-tag (red) and an HRV3C cleavage site (cyan) used for all smFRET and cryo-EM experiments (top). For determination of the oligomerization state using 2fFCS, we also created a construct with an additional cysteine at the N-terminus (bottom). Vertical line indicates the HRV3C cleavage position.

<table>
<tr><th>ComK amino acid sequence</th></tr>
<tr><td>MGSSHHHHHHSGSGSAGLEVLFQ|GPGMSQKTDAPLESYEVNGATIAVLPEEIDGKICSKIIEKDCVFYVNMKPLQIVDRSCRFFG
SSYAGRKAGTYEVTKISHKPPIMVDPSNQIFLFPTLSSTRPQCGWISHVHVKEFKATEFDDTEVTFSNGKTMELPISYNSFENQVY
RTAWLRTKFQDRIDHRVPKRQEFMLYPKEERTKMIYDFILRELGERY</td></tr>
<tr><td>MGSSHHHHHHSGSGSAGLEVLFQ|GPCMSQKTDAPLESYEVNGATIAVLPEEIDGKICSKIIEKDCVFYVNMKPLQIVDRSCRFFG
SSYAGRKAGTYEVTKISHKPPIMVDPSNQIFLFPTLSSTRPQCGWISHVHVKEFKATEFDDTEVTFSNGKTMELPISYNSFENQVY
RTAWLRTKFQDRIDHRVPKRQEFMLYPKEERTKMIYDFILRELGERY</td></tr>
</table>

Supplementary Table 4. Fitting parameters obtained with the mechanistic binding model and with the Hill equation Errors result from globaat least two independent experiments. To increase the robustness of the fit parameters in our mechanistic binding model, we fit the constructs in which we probed both boxes (*addAB* and *comG*) in global manner with the same parameter for both constructs. Fixed parameters are indicated by (*) and global fit parameters are indicated by (#).

Promoter	Δg_K (k_BT)	$-\Delta g_\sigma$ (k_BT)	$-\Delta g_J$ (k_BT)	K_{Hill} (nM)	n
comG: box 1 labelled	7.0 ± 0.6#	4.0*	4.5 ± 1.2#	15 ± 1	3.55 ± 0.12
comG: box 2 labelled	7.0 ± 0.6#	4.0*	4.5 ± 1.2#	16 ± 1	3.44 ± 0.27
comG: isolated box 1 labelled	6.1 ± 0.7	4.0 ± 0.7#	n.d.	73 ± 21	1.76 ± 0.02
comG: isolated box 2 labelled	5.7 ± 0.7	4.0 ± 0.7#	n.d.	48 ± 14	1.59 ± 0.09
comG GC-55%: box 1 labelled	6.6 ± 0.7	4.0*	3.7 ± 1.2	16 ± 1	2.97 ± 0.28
comG GC-72%: box 1 labelled	6.5 ± 0.5	4.0*	3.5 ± 0.9	17 ± 1	3.05 ± 0.08
comG Mismatch: box 1 labelled	6.1 ± 0.1	4.0*	3.1 ± 0.1	14 ± 1	2.93 ± 0.28
comG Nicked: box 1 labelled	4.2 ± 0.2	4.0*	0.2 ± 0.1	19 ± 8	1.82 ± 0.12
comK: box 1 labelled	5.8 ± 0.2	4.0*	1.9 ± 0.1	21 ± 2	2.28 ± 0.16
addAB1: box 1 labelled	8.0 ± 0.7#	4.0*	5.8 ± 0.9#	25 ± 6	4.34 ± 0.32
addAB2: box 2 labelled	8.0 ± 0.7#	4.0*	5.8 ± 0.9#	24 ± 7	3.77 ± 0.45
Design 8bp	6.7 ± 0.7	4.0*	4.1 ± 1.0	14 ± 2	3.85 ± 0.05
Design 14bp	5.8 ± 0.1	4.0*	1.4 ± 0.1	30 ± 5	2.44 ± 0.03
Design 18bp	6.8 ± 0.4	4.0*	4.8 ± 0.7	12 ±1	3.68 ± 0.09
Design 24bp	5.8 ± 0.6	4.0*	1.5 ± 0.9	28 ± 1	2.48 ± 0.39
Design 31bp	6.3 ± 0.3	4.0*	2.5 ± 0.5	25 ± 1	2.81 ± 0.07

Supplementary Table 5. Refinement statistics.

Structure	*addAB* (8 bp spacer)	*comG* (18bp spacer)
Space group	*P1*	*P1*
PDB ID		
Box		
Lengths (Å)	44.7,53.3,264.6	48.0, 48.0, 296.5
Angles (°)	90.0, 90.0, 90.0	90.0, 90.0, 90.0
Resolution Estimates (Å)		
d FSC model(0.143)	6.7	6.7
d FSC model(0.5)	10.0	10.2
CC (mask)	0.74	0.75
CC(box)	0.66	0.63
CC(peaks)	0.25	0.19
CC(volume)	0.73	0.75
Number of chains	2	2
Number of resolved nucleotides	156	168
Number of atoms	3198	3444
Bonds (RMSD)		
Bond (Å) ($> 4\sigma$)	0.004 (0)	0.005 (0)
Angle (°) ($> 4\sigma$)	0.570 (2)	0.665 (0)
All the information above was obtained from Phenix		

Supplementary Table 6. Comparison of the experimental mean transfer efficiencies with the simulated transfer efficiency of the *addAB* promoter. The best fit of the experimental FRET values is marked in red.

ComK Dimer Volume EM-map (nm^3)	Labelling position	Simulated smFRET values when allowing clashes of the first *N* linker atoms with DNA				Experimental smFRET values
		N = 0	*N* = 1	*N* = 4	*N* = 7	
19.6	Box1	n.d.	0.74	0.68	0.60	0.67 ± 0.03
	Box2	n.d.	0.54	0.38	0.30	0.40 ± 0.03
47.8	Box1	n.d.	0.75	0.68	0.60	0.67 ± 0.03
	Box2	n.d.	0.56	0.38	0.29	0.40 ± 0.03

n.d. : Simulations strictly considering all linker atom clashes (*N* = 0) do not allow the dye attachment.

Supplementary Table 7. DNA sequences used for gel retardation assay. The two AT boxes are highlighted in cyan. Only the forward strand is shown. The 18 bp spacer of the ComG promoter, was modified such that the spacer length was truncated or extended. To keep a uniform length for all dsDNA, the sequence length spanning the two AT boxes was altered, based on the genomic sequence, keeping the termini with a GC pair. Half-box constructs only contain one A-tract per box.

Promoter	Length	Sequence
**Spacer: 4 bp**	89	GCAGTTGAAAGTCTTTTTTCTTGCCAGAAAGAATTGGTTTTTCAGCAAAATCACGTTTTCCCTGTTTGATTACCTTTTCTTCTTTTTCG
**Spacer: 6 bp**	89	CAGTTGAAAGTCTTTTTTCTTGCCAGAAAGAATTGGTTTTTCAGCATAAAATCACGTTTTCCCTGTTTGATTACCTTTTCTTCTTTTTC
**Spacer: 8 bp**	89	CGTTGAAAGTCTTTTTTCTTGCCAGAAAGAATTGGTTTTTCAGCATATAAAATCACGTTTTCCCTGTTTGATTACCTTTTCTTCTTTTG
**Spacer: 10 bp**	89	GTTGAAAGTCTTTTTTCTTGCCAGAAAGAATTGGTTTTTCAGCATATAAAAAATCACGTTTTCCCTGTTTGATTACCTTTTCTTCTTTG
**Spacer: 12 bp**	89	CTGAAAGTCTTTTTTCTTGCCAGAAAGAATTGGTTTTTCAGCATATAACAAAAATCACGTTTTCCCTGTTTGATTACCTTTTCTTCTTG
**Spacer: 14 bp**	89	CGAAAGTCTTTTTTCTTGCCAGAAAGAATTGGTTTTTCAGCATATAACATCAAAATCACGTTTTCCCTGTTTGATTACCTTTTCTTCTG
**Spacer: 16 bp**	89	GAAAGTCTTTTTTCTTGCCAGAAAGAATTGGTTTTTCAGCATATAACATCTCAAAATCACGTTTTCCCTGTTTGATTACCTTTTCTTCG
**Spacer: 18 bp**	89	CAAGTCTTTTTTCTTGCCAGAAAGAATTGGTTTTTCAGCATATAACATCTCACAAAATCACGTTTTCCCTGTTTGATTACCTTTTCTTC
**Spacer: 20 bp**	89	CAGTCTTTTTTCTTGCCAGAAAGAATTGGTTTTTCAGCATATAACATCTCACCAAAAATCACGTTTTCCCTGTTTGATTACCTTTTCTG
**Spacer: 22 bp**	89	CGTCTTTTTTCTTGCCAGAAAGAATTGGTTTTTCAGCATATAACATCTCACCAGCAAAATCACGTTTTCCCTGTTTGATTACCTTTTCG
**Spacer: 24 bp**	89	GTCTTTTTTCTTGCCAGAAAGAATTGGTTTTTCAGCATATAACATCTCACCAGCATAAAATCACGTTTTCCCTGTTTGATTACCTTTTC
**Spacer: 26 bp**	89	TCTTTTTTCTTGCCAGAAAGAATTGGTTTTTCAGCATATAACATCTCACCAGCATATAAAATCACGTTTTCCCTGTTTGATTACCTTTT
**Spacer: 28 bp**	89	CTTTTTTCTTGCCAGAAAGAATTGGTTTTTCAGCATATAACATCTCACCAGCATATAAAAAATCACGTTTTCCCTGTTTGATTACCTTT
**Spacer: 30 bp**	89	TTTTTTCTTGCCAGAAAGAATTGGTTTTTCAGCATATAACATCTCACCAGCATATAACAAAAATCACGTTTTCCCTGTTTGATTACCTT
**Spacer: 32 bp**	89	TTTTTCTTGCCAGAAAGAATTGGTTTTTCAGCATATAACATCTCACCAGCATATAACATCAAAATCACGTTTTCCCTGTTTGATTACCT
**Spacer: 34 bp**	89	TTTTCTTGCCAGAAAGAATTGGTTTTTCAGCATATAACATCTCACCAGCATATAACATCTCAAAATCACGTTTTCCCTGTTTGATTACC
**Spacer: 36 bp**	89	TTTCTTGCCAGAAAGAATTGGTTTTTCAGCATATAACATCTCACCAGCATATAACATCTCACAAAATCACGTTTTCCCTGTTTGATTAC
**Half box Δ14**	89	CAAGTCTTTTTTCTTGCCAGAACGCGTTGGTTTTTCAGCATATAACATCTCACAAAATCACGCGCGCCCTGTTTGATTACCTTTTCTTC
**Half box Δ23**	89	CAAGTCTTTTTTCTTGCCAGAAAAAATTGGTCGCGCAGCATATAACATCTCACCGCGTCACGTTTTCCCTGTTTGATTACCTTTTCTTC
**Half box Δ24**	89	CAAGTCTTTTTTCTTGCCAGAAAAAATTGGTCGCGCAGCATATAACATCTCACAAAATCACGCGCGCCCTGTTTGATTACCTTTTCTTC
**Half box Δ13**	89	CAAGTCTTTTTTCTTGCCAGAACGCGTTGGTTTTTCAGCATATAACATCTCACCGCGTCACGTTTTCCCTGTTTGATTACCTTTTCTTC
