# Supplementary material for: Allostery through DNA drives phenotype switching
Source: Nat Commun. 2021 May 20;12:2967. doi: 10.1038/s41467-021-23148-2 (PMC8170675; doi:10.1038/s41467-021-23148-2)
Supplement: Supplementary file 2 — Reporting Summary [file 41467_2021_23148_MOESM2_ESM.pdf]

nature research

Corresponding author(s): Gabriel Rosenblum
Hagen Hofmann

Last updated by author(s): Mar 13, 2021

Reporting Summary

Nature Research wishes to improve the reproducibility of the work that we publish. This form provides structure for consistency and transparency in reporting. For further information on Nature Research policies, see our Editorial Policies and the Editorial Policy Checklist.

Statistics

For all statistical analyses, confirm that the following items are present in the figure legend, table legend, main text, or Methods section.

n/a	Confirmed	
☐	☒	The exact sample size (n) for each experimental group/condition, given as a discrete number and unit of measurement
☐	☒	A statement on whether measurements were taken from distinct samples or whether the same sample was measured repeatedly
☒	☐	The statistical test(s) used AND whether they are one- or two-sided<br>*Only common tests should be described solely by name; describe more complex techniques in the Methods section.*
☒	☐	A description of all covariates tested
☒	☐	A description of any assumptions or corrections, such as tests of normality and adjustment for multiple comparisons
☐	☒	A full description of the statistical parameters including central tendency (e.g. means) or other basic estimates (e.g. regression coefficient) AND variation (e.g. standard deviation) or associated estimates of uncertainty (e.g. confidence intervals)
☒	☐	For null hypothesis testing, the test statistic (e.g. F, t, r) with confidence intervals, effect sizes, degrees of freedom and P value noted<br>*Give P values as exact values whenever suitable.*
☒	☐	For Bayesian analysis, information on the choice of priors and Markov chain Monte Carlo settings
☒	☐	For hierarchical and complex designs, identification of the appropriate level for tests and full reporting of outcomes
☒	☐	Estimates of effect sizes (e.g. Cohen's d, Pearson's r), indicating how they were calculated

Our web collection on statistics for biologists contains articles on many of the points above.

Software and code

Policy information about availability of computer code

Data collection	Data collection software was done using PicoQuant SymphoTime 64 (Version 2.1.3813) for the single-molecule FRET data. Data collection for cryo-EM data was performed with the software SerialEM.
Data analysis	We used a custom module for Mathematica 11.2 (Wolfram Research) for the analysis of single-molecule fluorescence data, which is available at https://schuler.bioc.uzh.ch/programs/. The cryo-EM data were analyzed using a combination of CryoSPARC 2.14.2. Visualization of the EM-data were done with the softwares UCSF Chimera (Version 1.12) or ChimeraX (Version 0.92).

For manuscripts utilizing custom algorithms or software that are central to the research but not yet described in published literature, software must be made available to editors and reviewers. We strongly encourage code deposition in a community repository (e.g. GitHub). See the Nature Research guidelines for submitting code & software for further information.

Data

Policy information about availability of data

All manuscripts must include a data availability statement. This statement should provide the following information, where applicable:

- Accession codes, unique identifiers, or web links for publicly available datasets
- A list of figures that have associated raw data
- A description of any restrictions on data availability

Cryo-EM maps and atomic coordinates have been deposited in the Electron Microscopy Data Bank (EMDB) and Protein Data Bank (PDB). The accession codes are EMD-11022 and 6Z0S (https://doi.org/10.2210/pdb6Z0S/pdb) for the comG-ComK complex (18 bp spacer) and EMD-12260 and 7NBN (https://doi.org/10.2210/pdb7NBN/pdb) for the addAB-ComK complex (8 bp spacer). The single-molecule raw data were deposited on the Zenodo data repository under the doi: 10.5281/zenodo.4468372. Data supporting the findings of this manuscript are available from the corresponding authors upon reasonable request. The source data underlying mean values and gel images in Figure panels 2a, 2b, 2d, 3b, 4b, 4c, 4d, 4e, 4f, S1a, S1b, S1c, S3a, S3b, S9 are provided as a Source Data file.

Field-specific reporting

Please select the one below that is the best fit for your research. If you are not sure, read the appropriate sections before making your selection.

- [x] Life sciences
- [ ] Behavioural & social sciences
- [ ] Ecological, evolutionary & environmental sciences

For a reference copy of the document with all sections, see nature.com/documents/nr-reporting-summary-flat.pdf

Life sciences study design

All studies must disclose on these points even when the disclosure is negative.

Sample size	The sample size for free diffusion measurements was always > 1000 bursts. The sample size was determined using the burst identification algorithm detailed in the Methods section.
Data exclusions	Bursts from molecules with only a single fluorophore and with < 80 photons were excluded from analysis to minimize the contribution of shot-noise in the FRET histograms.
Replication	The overall reproducibility of the experiments was confirmed by multiple measurements. The sample size used to determine derived parameters such as Hill exponents are indicated.
Randomization	No randomization was used.
Blinding	Single-molecule experiments were not blinded.

Reporting for specific materials, systems and methods

We require information from authors about some types of materials, experimental systems and methods used in many studies. Here, indicate whether each material, system or method listed is relevant to your study. If you are not sure if a list item applies to your research, read the appropriate section before selecting a response.

Materials & experimental systems

n/a	Involved in the study	
x		Antibodies
x		Eukaryotic cell lines
x		Palaeontology and archaeology
x		Animals and other organisms
x		Human research participants
x		Clinical data
x		Dual use research of concern

Methods

n/a	Involved in the study	
x		ChIP-seq
x		Flow cytometry
x		MRI-based neuroimaging
